# Physical One‐Way Functions for Decentralized Consensus Via Proof of Physical Work

**DOI:** 10.1002/advs.202409386

**Published:** 2024-12-16

**Authors:** Marvin Winkler, Catharina Peither, Simon Petrick, Lothar Seidemann, Holger Jelich, Frank Kleine Jäger, Jörn Müller‐Quade, Alexander Colsmann, Hermann Nirschl, Frank Rhein

**Affiliations:** ^1^ Institute of Mechanical Process Engineering and Mechanics (MVM) KIT Strasse am Forum 8 76131 Karlsruhe Germany; ^2^ Material Research Center for Energy Systems (MZE) KIT Strasse am Forum 7 76131 Karlsruhe Germany; ^3^ Light Technology Institute (LTI) KIT Engesserstrasse 13 76131 Karlsruhe Germany; ^4^ BASF SE Carl‐Bosch‐Strasse 38 67056 Ludwigshafen/Rhein Germany; ^5^ Institute of Information Security and Dependability (KASTEL) KIT Am Fasanengarten 5 76131 Karlsruhe Germany

**Keywords:** cryptography, decentralized consensus, physical one‐way function, proof of work

## Abstract

Decentralized consensus on the state of the Bitcoin blockchain is ensured by proof of work. It relies on digital one‐way functions and is associated with an enormous environmental impact. This paper conceptualizes a physical one‐way function that aims to transform a digital, electricity‐consuming consensus mechanism into a physical process. Boundary conditions for the security requirements are established and discussed as well as experimentally investigated for a specific setup based on printing and optical analysis of pigment‐carrier composites. In the context of the applied methods, this setup promises to be mathematically unclonable, steady, reproducible, collision resistant and non‐invertible and illustrates the feasibility of a physical one‐way function. Based on this, a framework for proof of physical work is conceptualized, which has the potential of a drastically lower CO_2_ footprint. This work initiates a progressive, interdisciplinary field of research and demands further investigations with regards to alternative setups, security definitions and strategies for challenging them.

## Graphical abstract

The novel concept of a physical one‐way function is presented, which promises a significant CO_2_ reduction for decentralized consensus mechanisms such as the proof of work in Bitcoin. Security requirements are discussed and experimentally investigated in a system based on printing and optical analysis of pigment structures.

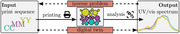

## 1 Introduction

**Figure 1:**
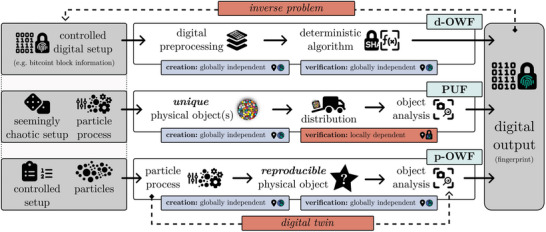
Illustration of a digital one‐way function (d‐OWF, top), physical unclonable function (PUF, middle) and physical one‐way function (p‐OWF, bottom). Dashed lines mark unwanted paths to either inverse the function or to bypass the physical aspect of a p‐OWF with a digital twin.

In an era that highly relies on digital interactions, cryptography and blockchain technology play a pivotal role in shaping a trustworthy and transparent future. Digital one‐way functions (d‐OWF), exemplified by cryptographic hash functions like SHA‐256,^[^ ^1^ ^]^ form the backbone of secure communications and decentralized ledgers. In essence, they quickly provide a deterministic output for any given input, while it is impossible to find a corresponding input for any given output (inverse problem). The concept is visualized at the top of **Figure** **1**. A prominent application example of SHA‐256 is the cryptocurrency Bitcoin,^[^ ^2^ ^]^ where all ever‐made transactions are stored in a public blockchain. Consensus on which transactions are added to the blockchain is reached without a central institution by the so‐called proof of work (PoW). To add a new block, participants of the peer‐to‐peer network compete to find solutions for specific inverse problems of SHA‐256. This is commonly referred to as mining and can only be done by trial and error. Once a solution is found and shared across the network, the design of the d‐OWF allows anyone, independent of their location, to readily verify its integrity. The successful miner receives a reward, which keeps the participants economically motivated to participate. The blockchain is trustworthy, because all blocks are unambiguously connected and hence, rewriting the blockchain would require a malicious individual to find multiple solutions in competition with the entire network, which is an unfeasible amount of work. A crucial downside of this PoW system is that it requires computational power (work) on a massive scale: In December 2017, the peak power demand of the Bitcoin network was between 1.3 and 14.8 GW, which is similar to the installed capacities of Finland and Denmark.^[^ ^3^ ^]^ The global carbon footprint of the network was estimated at 65.4 Mt CO_2_ per year in 2021 which is comparable to the country‐level emissions of Greece (56.6 Mt CO_2_ in 2019), representing 0.19 % of the global CO_2_ emissions.^[^ ^4^ ^]^

The physical world is full of complex phenomena that cannot be fully described by analytical equations, like e.g. the family of so‐called *n*‐body problems in physics^[^ ^5^ ^]^ or the interactions of granular materials, i.e. heterogeneous particle assemblies with light.^[^ ^6^ ^]^ This motivates the search of physical analogies for digital cryptographic functions. An established and standardized example are physical unclonable functions (PUF). In general, a PUF is a physical object that is statistically improbable to occur twice as well as hard to reproduce digitally but behaves deterministically based on its specific properties.^[^ ^7^ ^]^ This means that when given the same input or so‐called challenge repeatedly, it yields the same response. They are mostly based on electrical circuits,^[^ ^8^ ^]^ but can also be designed by chemical^[^ ^9^ ^]^ or biological^[^ ^10^ ^]^ methods, the interaction of light with matter^[^ ^11^ ^]^ or be as simple as colored candy patterns.^[^ ^12^ ^]^

PUFs are mostly used in anti‐counterfeiting applications, where ownership and authenticity are proven by validating certain challenge‐response pairs against a central database. Figure 1 visualizes the basic concept of a PUF based on ref. [12]. A seemingly chaotic particle process creates a unique candy particle assembly. Any specific process outcome is impossible to predict and cannot be reproduced. A key difference to d‐OWFs is the fact that only the owner of a specific, singular PUF can verify its digital fingerprint locally. By definition, a truly decentralized PoW system cannot be built around a singular object or central source of trust. Hence, PUFs are no physical alternative to d‐OWFs.

This study aims at bridging this gap by presenting the novel concept of physical one‐way functions (p‐OWF), which are a true physical counterpart to d‐OWFs and are illustrated in Figure 1 (bottom). Given a set of parameters as input, a controlled process generates a reproducible physical object, which is analyzed in a defined way to yield the output. In contrast to PUFs, p‐OWFs are physical processes, not objects. As indicated in Figure 1, both the creation and verification must be globally independent, which is another key difference to PUFs. The output must be deterministic, but not easily linked back to the input, i.e. the inverse problem must still be hard to solve. In other words, knowing how to produce a physical object that produces a certain output should require trial and error, i.e. physical work. Furthermore, the series of operations must not be replaceable by a digital twin, as this would otherwise result in the p‐OWF being reverted to a digital one.

An example of a p‐OWF based on the complex interactions of light with pigment‐substrate composite structures is presented. It is discussed how this p‐OWF can address the environmental impact of current PoW systems. Subsequently, the security requirements are discussed and investigated experimentally for the proposed system. Finally, a decentralized proof of physical work (PopW) concept is presented to showcase the potential applications for p‐OWFs. Note that this study does not claim to present a ready‐to‐launch PopW system, but rather exemplify the possibilities and lay the foundation for future work in this uncharted field of research. It aims at sparking ideas for other p‐OWF setups from all scientific fields and provides guidelines on which properties are essential and how to assess them.

## 2 Results

### 2.1 Security Requirements, Benefits and Setup for a Physical One‐Way Function

To illustrate the security requirements with a specific example, this paper follows the hypothesis that the process of applying dye molecules onto defined substrate structures and measuring the interaction of this composite with light can be considered a p‐OWF. In short, cross‐linked dye molecule clusters form pigment particles with characteristic absorption bands. Moreover, the particle size and distribution on the substrate defines the shape of the measured spectrum which typically alters the color perception of a print.^[^ ^13^ ^]^ Both UV/vis extinction and reflection are considered as complex physical mechanisms and their usage is explained in detail below.

In the following, the presented molecular dyes are generally referred to as pigments that are printed on two different kinds of substrates with porous surface coatings. During this process, layered pigment particle structures in the nanometer range are created inside those coatings. More detailed information on dye characteristics, substrate surface and the structure of the final dye‐carrier composite is presented and discussed in the Supporting Information. Knowing this, the input to the considered p‐OWF consists of a sequence of defined amounts of different pigments printed on top of each other. In this work, an off‐the‐shelf ink‐jet printer is used with the three possible pigment types cyan, magenta and yellow. Light in the visible range is directed at this particle structure and either the reflectance or extinction is measured. In this system, the cost per evaluation is tied to physical resources (pigments) and time instead of electricity. During PoW, a certain reward is offered and hence, the invested resources are economically limited. Certain physical resources, but time most prominently, are known to produce less CO_2_ per USD of economic value than electricity, hence reducing the environmental footprint of the PopW system. An exemplary calculation is given in the Discussion S1 (Supporting Information) to illustrate this point.

The general security requirements for a p‐OWF are summarized below and it is described how they relate to the proposed setup. Although similar, the requirements differ significantly from the standardized PUFs^[^ ^7^ ^]^ in particular with respect to reproducibility. Additionally, the proposed p‐OWF is not a physical object, but rather the physical process of producing an object and performing a defined action with it.

1) **Mathematical Unclonability**: There must be no digital bypass or substitute for the physical procedure. This can be considered a weak criterion, as if non‐invertibility still holds, this results in a digital one‐way function.
  The layering of particles can generally be simulated numerically using the discrete element method (DEM)^[^ ^14^ ^]^ and the interaction of light with solid particles is described by Maxwell's equations that can be solved analytically for simple geometries.^[^ ^6^ ^]^ However, the behavior of real‐world particles is complex, as they possess multidimensionally distributed properties like shape and size.^[^ ^15^ ^]^ Additionally, particles in a collective are electromagnetically coupled and when the concentration is high, multiple scattering occurs, making the solution of Maxwell's equations hard.^[^ ^6^ ^]^ We therefore state that the setup is mathematically unclonable, as the effort required would far surpass performing the actual experiments and the achievable accuracy is strongly limited to the complex behavior of particles.
2) **Non‐Invertibility**: Given an output, there must be no substantially better way of finding a corresponding input than trial and error.
  Going from a given spectroscopic measurement back to the actual particle structure, i.e. the inverse problem in spectroscopy, is hard^[^ ^6^ ^]^ and not analytically solvable due to the reasons discussed above. If it was analytically solvable, spectroscopy would no longer require elaborate calibration measurements as detailed sample information could be readily calculated from obtained spectra. However, this does not guarantee that no data‐driven or empirical solution to this inverse problem exists. By measuring enough input‐output pairs, machine learning algorithms might indeed be able to make this connection. These so‐called modeling attacks are actively researched in the scope of PUFs^[^ ^16^, ^17^ ^]^ and investigated in a later section.
3) **Collision Resistance**: It should be unlikely for any two distinct inputs to produce the same output, which is defined as a collision.
  Different pigment structures produce different spectra, which is what we generally perceive as the “color” of a print. Nevertheless, the setup is definitely not collision‐free on the entire input range. For example, if the amount of pigments increases toward infinity and extinction is measured, after a certain point, all light will be extinct (black) and the actual pigment structure and sequence will be unimportant. Note that the existence of collisions does not make a p‐OWF generally unsuitable for the use in PopW: More collisions make the inverse problem easier, which might be balanced by increasing the overall PopW difficulty.
4) **Steadiness**: Analogously to a PUF, a p‐OWF must quickly and deterministically produce a certain output for a certain input.
5) **Reproducibility**: In contrast to a PUF, this input‐output relationship must be confirmable by any person independent of location.
  Reproducibility requires that anyone with the defined materials and setup is able to print a given sequence (input) and obtain the same physical structure. Analogously to PUFs, steadiness requires that the optical analysis of this structure is then reproducible, i.e. that repeated measurements yield the same spectra. Thus, reproducibility and steadiness are tied together and describe the experimental and analytical errors. For simplification, both requirements are summed up under the term reproducibility for the remainder of the article. It is self‐evident that perfect reproducibility cannot be achieved in the physical world, as there will always be uncertainties and imperfections involved. However, a physical process might indeed be reproducible in a certain window of accepted results. This means that reproducibility, i.e. the experimental deviations, are closely linked to the number of collisions, i.e. what we consider an “identical” output. Reproducibility is therefore linked to collision resistance and experimentally investigated in the following section.

### 2.2 Experimental Analysis of Reproducibility and Collision Resistance

The cryptographic hash function SHA‐256 is deterministic and practically collision‐free, meaning that any change of the input is significant to the output. The process of hashing is based on bitwise operations made on binary encoded messages and therefore innately resistant to error.^[^ ^1^ ^]^ When dealing with physical systems, two major sources of error come to mind: First, sample preparation (printing) will vary between repetitions, due to inhomogeneities in the ink and topological differences of the substrate. Second, errors in the extinction or reflection measurement can occur due to inaccuracies of the measurement instrument itself. Therefore, going from a digital printing sequence like

(1) $$\begin{array}{ccc} & & 1.\;C\;\to \;2.\;C\;\to \;3.\;M\;\to \;4.\;M\;\to \;5.\;Y\;\to \\ & & \;6.\;Y\;(\text{short: CCMMYY, input}) \end{array}$$

to a measured spectrum (output), will include certain deviations and not be perfectly unique. With this in mind, a collision (i.e. same output for different inputs) occurs, when spectra resulting from different print settings are not significantly different, i.e. when their values fall within the respective statistical confidence intervals. Vividly, both spectra do not convey a unique message or information and can be considered “identical.”

**Figure 2:**
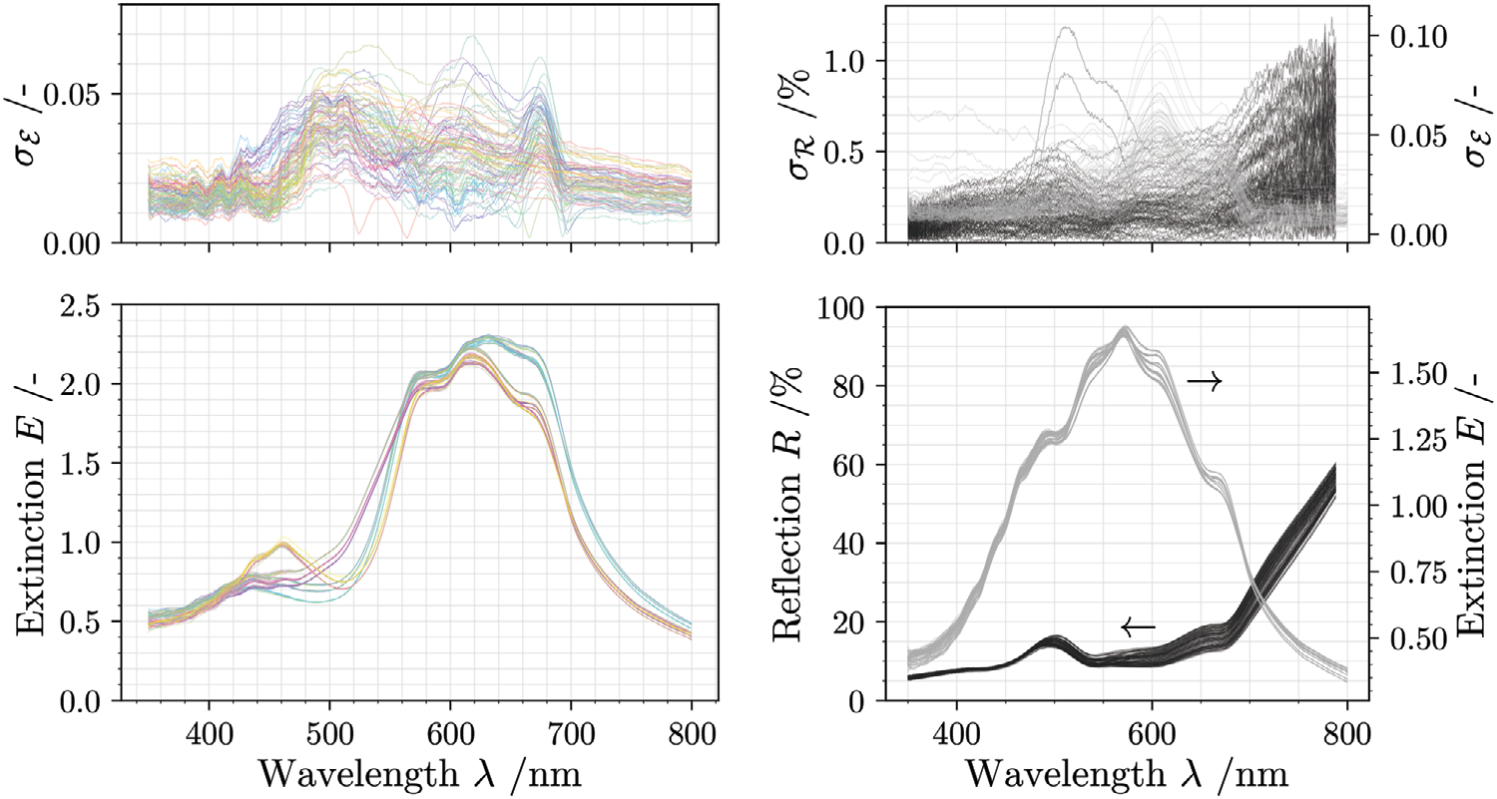
Measured optical signals at multiple wavelengths for both sample set 4L‐P81‐E (left) and {6L‐PM90‐E, 6L‐PM90‐R} (right). The standard deviation from the triplicate measurement is given above each plot. Samples are colored based on the total amount of cyan magenta and yellow (perceptible color). For six layers, all samples appear grayish.

The Experimental Section provides details on the experimental procedure and design, while a more detailed analysis is provided in the Supporting Information. In general, three data sets were produced: A four‐layer (4L) full permutation (P) set, consisting of 81 sequences (81) measured with extinction (E), 4L‐P81‐E and two six‐layer (6L) permutation multisets (PM, 2C, 2M, 2Y), consisting of 90 sequences each (90) measured with extinction 6L‐PM90‐E and reflectance (R) 6L‐PM90‐R. Each sequence was printed in triplicate and each sample was measured ten‐fold. Within each set, all measurements of a sequence were averaged and are represented by a matrix $\bar{E}$ or $\bar{R}$, while the standard deviation matrices are given by $\sigma_{E}$ and $\sigma_{R}$. All measured optical signals for each individual sequence and their evaluated standard deviations are shown in **Figure** **2**. To identify collisions, all samples are iteratively compared, resulting in the respective amount of index pairs {i,j} of the strictly upper triangular matrix. Following this strategy, the wavelength‐dependent overlap

(2) $$\chi_{i,j}^{E}\left(\lambda \right)=\left\{\begin{array}{cc} 1, & \text{if}\;\;\;\;\;|{\bar{E}}_{i}\left(\lambda \right)-{\bar{E}}_{j}\left(\lambda \right)|\leq \frac{1}{2}\left(\sigma_{i}^{E}\left(\lambda \right)+\sigma_{j}^{E}\left(\lambda \right)\right)S\\ 0, & \text{otherwise} \end{array}\right.$$

for extinction datasets E can be calculated. $\chi_{i,j}^{R}\left(\lambda \right)$ for the reflection dataset R follows analogously. A noise factor *S* introduces artificial uncertainties into the analysis and is later used to perform a sensitivity analysis. A collision event is defined by

(3) $$\zeta_{i,j}=\left\{\begin{array}{cc} 1, & \text{if}\;\;\;\;\;{\vec{\chi }}_{i,j}=\vec{1}\\ 0, & \text{otherwise}\;\;\;\;\;\;\; \end{array}\right.$$

and true, if both spectra overlap at every wavelength. The final evaluation parameter describes the collision probability

(4) $$Z=\frac{2}{P(P-1)}\;\left[\sum_{i=1}^{P}\sum_{j=i+1}^{P}\zeta_{i,j}\right]$$

in the analyzed permutation set of spectral data, where *P* is the total number of sequences.

**Figure 3:**
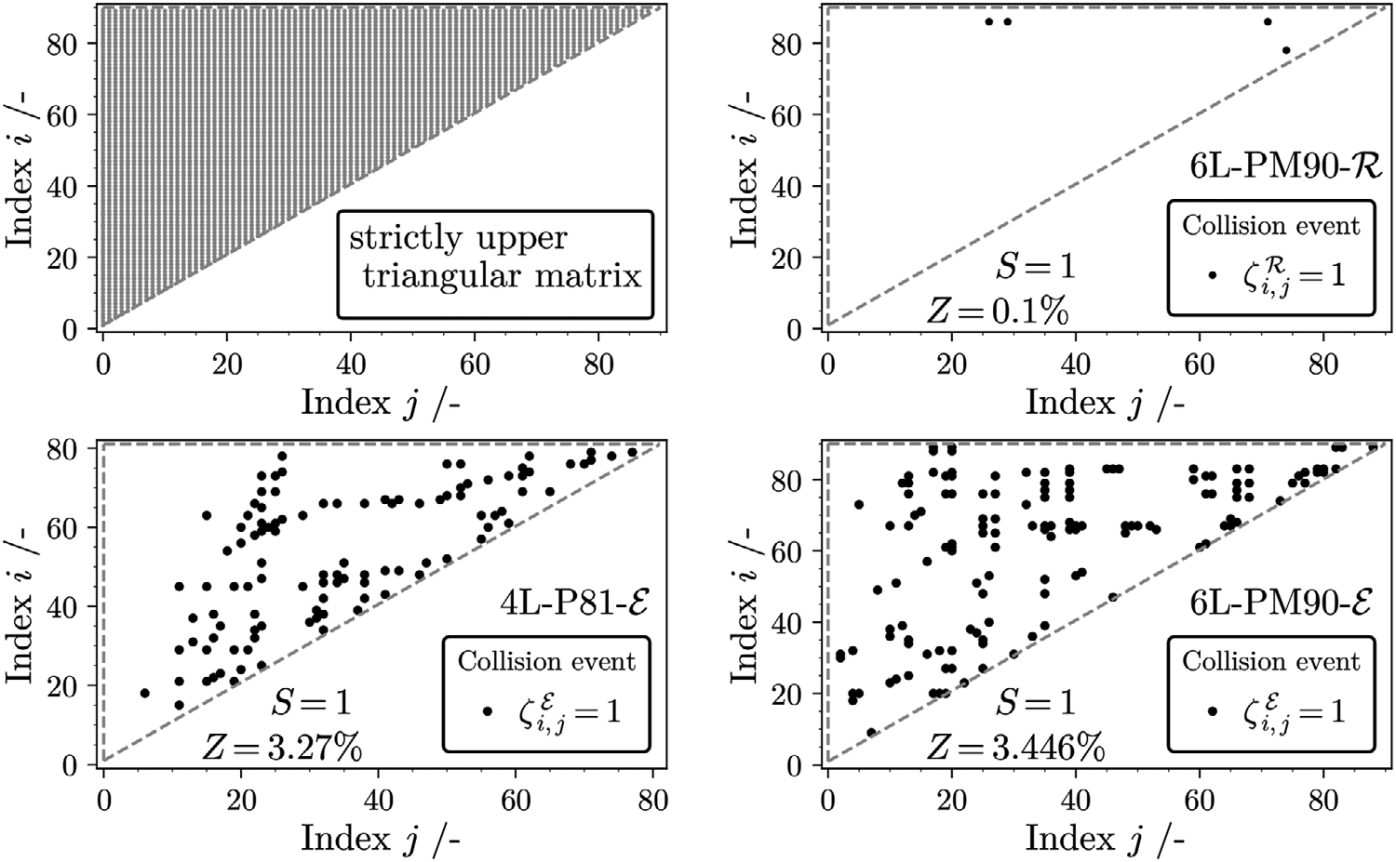
Illustration of the strictly upper triangular matrix for each sample index pair (*i*, *j*) (top left) and collision events for each of the three analyzed permutation sets (top right, bottom left and right). The average collisions per sample *Z* and applied safety factor *S* are shown as annotations in the respective plots.

**Figure** **3** shows the upper triangular matrix on the top left with all possible index pairs marked as small gray dots. The other sub‐figures on the top right and bottom highlight all registered collision events according to equations 2 and 3. The flat noise factor was set to *S* = 1 in all cases. As an additional evaluation measure, the collision probability *Z* is displayed. It can be stated, that the reflection data set (6L‐PM90‐R) shows a remarkable yet imperfect significance. At closer inspection, however, this imperfection boils down to two sequences with index *i* = 78 and *i* = 86. The latter is responsible for three collisions that can be attributed to human error or unknown circumstances during printing. A larger mean standard deviation of the reflection measurement compared to the other samples supports this statement and is highlighted in Figure S5 (Supporting Information). The results suggest that the reflection measurement of pigment structures with a six‐layer complexity is reproducible and yields a collision probability of only 0.1 %. In comparison, for both 4L‐P81‐E and 6L‐PM90‐E the collision probability *Z* is greater than three percent. This implies that multiple sequences could potentially result in the same spectrum within the measured confidence interval. It should be noted that this might be attributed to non‐homogeneity of the translucent substrate. The fact that light first needs to pass through the thin foil reduces the overall signal strength and therefore the signal‐to‐noise ratio. This leads to a higher standard deviation between printed samples compared to the reflection dataset utilizing high‐quality paper. These findings indicate that, based on both experimental setups used in this study, the measurement of reflected light is less error‐prone. In a practical application of the p‐OWF during PopW, this implies that finding a valid printing sequence (input) for a given spectrum (output) is harder in the reflectance‐based system. Additionally, for every network participant to be able to validate a found solution with defined certainty, the range of accepted outputs can be lower. Note that the extinction measurement setup used in this work could still be optimized in further research to reduce the standard deviation. The statement that dataset 6L‐PM90‐R is more suitable for the proposed p‐OWF is strictly applicable only in the context of this study and the measurement systems used.

**Figure 4:**
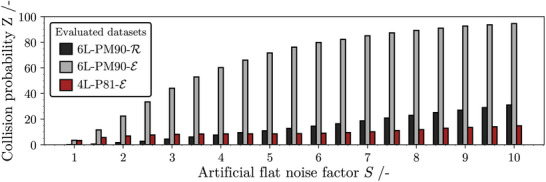
Evolution of collision probability *Z* with increasing flat noise factor *S*, which favors the overlap of confidence intervals of spectra pairs during iterative comparison.

To address the sensitivity of the collision probability to an increasingly non‐ideal measurement, the calculation of *Z* is repeated for different values of *S* and the results are compared and visualized in **Figure** **4**. *Z* increases with the artificial increase of the standard deviation for every data set. A closer inspection of Figure 2 reveals that extinction spectra of samples with identical amounts of pigments but varying sequence tend to have a similar shape whereas different amounts of pigments result in different perceptible “colors” and draw a different spectral shape. This statement is trivial in terms of colorimetry, but is important to mention here, because similar shapes raise the probability of a collision event. Since only certain sequences within the complete permutation set 4L‐P81‐E contain the same pigment distribution, collisions are not as sensitive to a flat increase of the standard deviation by *S*. In contrast, the collision probability of the extinction data in the permutation multiset 6L‐PM90‐E rapidly approaches 100 %. The identical sequences, printed on paper (6L‐PM90‐R), show a lower tendency toward collision, even if the standard deviation is artificially increased. This can be explained by the non‐uniform influence of each layer on the reflection spectrum, a dependence that varies with the height of each layer. Specifically, the uppermost layers within the stack exert a more pronounced influence on the amount of reflected light, which is further discussed in the following section. In other words, reflection spectroscopy is more adept at capturing the “color” attributes of the top layer, contributing to an increased variance in the spectral shape and enhancing collision resistance. Images taken with a laser scanning microscope of selected samples, as shown in Figure S2 (Supporting Information), support this assumption.

### 2.3 Experimental Analysis of Non‐Invertibility

**Figure 5:**
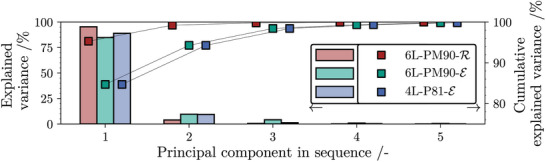
Bar chart showing proportion of variance explained by the first five principle components (PCs) in all three analyzed datasets. Scattered points indicate the cumulative variance explained by the transformed input data.

A p‐OWF must be non‐invertible, i.e. given a specific spectrum (output), there must be no analytical or data‐driven model to accurately predict the pigment sequence (input). The first step is to analyze the spectroscopic raw data and try to combine and reduce the number of measured features while retaining as much information as possible. Based on this, a grouping of all samples can be conducted which aims to facilitate a more physics‐based evaluation of internal dependencies. Moreover, the subsequent training of data‐driven models is accelerated when they are trained on a smaller number of more relevant input parameters.^[^ ^18^ ^]^ Therefore, a principal component analysis (PCA) is performed and the initial complexity of all three datasets are reduced to *c* = 5 components each, resulting in the following transformations: $\bar{E}\to X_{E}$ and $\bar{R}\to X_{R}$. During calculation, it is ensured that the transformed optical signals are uncorrelated and have zero unit variance. This form of pre‐processing is called whitening and can be beneficial when using the PCA output in downstream modeling.^[^ ^19^ ^]^ The so‐called scree plot in **Figure** **5** visualizes the percentage of explained variance per principal component alongside the cumulative variance. It is apparent that the first PC describes most of the signal variability for all three datasets. Additionally, five principle components are sufficient to raise the cumulative explained variance above 98 % in each case, i.e. only a minimal amount of information was lost during dimensionality reduction.

**Figure 6:**
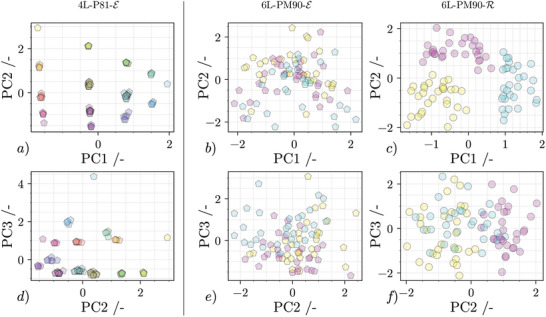
Score plots of transformed spectroscopic data by principle component analysis (PCA). Top row shows scoreplot of first and second principal component (PC), bottom row shows scoreplots of second and third PC. Each column is associated with one dataset. In subfigure *a* and *d*, each sample is represented by a point colored with the samples perceptible color. In subfigure *b*,*c*,*e* and *f* each sample is colored after the pigment type of the last layer.

Even before the transformed data is used for training a data‐driven model, its now condensed state, combined with a score plot, allows a clear and qualitative observation of intrinsic patterns. **Figure** **6** visualizes the principal component combinations PC1 and PC2, as well as PC2 and PC3 for the respective data sets in three subfigure columns. Each point in a subfigure row represents a unique sequence of the original dataset. A predominant spatial grouping, as it can be seen e.g. in Figure 6*a*, reveals similarities between samples. In many cases, if prior knowledge of sample features are known, the grouping can be further supported, clearly visualized or even first brought to light by a referenced coloration of each individual point. Two different types of coloring have been applied to the data in Figure 6. Focusing on the first column (Figure 6*a*,*d*), samples are colored based on the total amount of each pigment type and the resulting mixed RGB color. As an example, the sample CCCC is colored in pure cyan whereas the sample CCYY and YYCC are colored in green. Both score plots indicate that grouping by pigment quanta is very simple. This suggests that a data‐driven model is very likely to make good predictions on the amount of each pigment, i.e. overall “color,” when presented with unseen data. Regarding the two data sets which are based on a complexity of six layers, this statement is not applicable. Since *P*_M_ is a permutation multiset, each and every sample has the identical amount of each pigment type and only the print sequence introduces relative variance between these spectra. It was theorized in the previous section that the uppermost layers have a more pronounced impact on the resulting spectra. To investigate this, the points in columns two (Figure 6*b*,*e*) and three (Figure 6*c*,*f*) are colored with either cyan, magenta or yellow depending on the pigment that was printed last. Figure 6*c* shows a clear spatial grouping of the sample points in the first two score plots for 6L‐PM90‐R, indicating the dominant influence of the last layer. In comparison, the same coloration applied to transformed data emerging from extinction measurements allows for no consistent grouping by human observation. Regarding basic principles of illumination and light interaction it is obvious that the top layers of pigment have a greater influence when their reflected light is measured. During an extinction measurement, however, every pigment layer interacts with the incident light waves.

The conducted pattern analysis by PCA poses legitimate doubts as to whether the presented concept of a p‐OWF is genuinely save to use in a PopW framework. Although it does not directly yield the corresponding sequence to a given spectrum, the PCA gives hints on how to approach the inverse problem. Building on this, the vulnerability of the proposed p‐OWF against modeling attacks is investigated and quantified by training of data‐driven models that predict pigment sequence (input) based on the measured spectra or their equivalent PCs (output). Note that there is an unlimited amount of applicable model architectures that will perform differently on this inverse problem. Since it is impossible to test all of them, it is generally impossible to prove non‐invertibility, although finding one well‐performing model will directly prove its non‐existence. A vivid example is ref. [17] inverting the proposed PUF by ref. [8] with a novel, tailor made ML‐algorithm although non‐invertibility was thoroughly tested during the original publication.

Supervised classification is a technique that uses labeled datasets to train algorithms that can accurately assign instances (samples) to predefined classes. Decision tree classifiers (DTC) are a popular subset of these methods, as they offer a clear and transparent hierarchical model structure and deterministic training procedure.^[^ ^20^, ^21^ ^]^ It is argued that an initial study on non‐invertibility should therefore be performed with such a transparent model, as it might link back to the weaknesses of the p‐OWF and help to increase resistance in future work. Additionally, the discrete nature of the predictions fits well with the approach of printing discrete pigment quanta. Investigations on data‐driven inversion are performed with a supervised DTC model included in the Python module “scikit‐learn.”^[^ ^22^ ^]^ Several parallel classification models (I − VI) are trained to predict the pigment type in a specific layer. For this, all samples have to be assigned a class label encoded by a numerical value as shown in **Table** **1**.

**Table 1:** Label encoding of permutation sets 4L‐P81‐E, 6L‐PM90‐E and 6L‐PM90‐R used for pattern analysis and data driven inversion of print settings. Few examples explain the basic principle of sample labeling for six individual data driven models (I–VI).

Sample	Encoding by layer selection					
↓	■□…□□	□■…□□	■■…□□	□□…■■	□□…■□	□□…□■
CCCC	0	0	0	0	0	0
CCCM	0	0	0	1	0	1
⋮	⋮	⋮	⋮	⋮	⋮	⋮
YYYY	2	2	8	8	2	2
CCMMYY	0	0	0	8	2	2
CMCMYY	0	1	1	8	2	2
⋮	⋮	⋮	⋮	⋮	⋮	⋮
YYMMCC	2	2	8	0	0	0
Model →	I *first*	II *second*	III *first two*	IV *last two*	V *penultimate*	VI *last*
Vector →	${\vec{y}}_{\;I}$	${\vec{y}}_{\;\mathrm{II}}$	${\vec{y}}_{\;\mathrm{III}}$	${\vec{y}}_{\;\mathrm{IV}}$	${\vec{y}}_{\;V}$	${\vec{y}}_{\;\mathrm{VI}}$

The assessment of the prediction accuracy for each model is performed with a defined evaluation routine: First, the input matrix X and each associated response vector $\vec{y}$ are split into a train and test subset. The train subset is used to train six DTC models, one for each column of Table 1. These models are used to predict the pigment type(s) in the corresponding layer(s) for the *test* subset, which are compared to their true values. Due to the discrete nature of the dataset, a prediction (classification) can only be either correct or incorrect. The total number of correct classifications is then divided by the test data size, giving a success rate between 0 % and 100 %. This procedure is repeated 100 times for random splits of training and test data. This allows for more robust statistics and prevents predictive performance from being affected by a biased or coincidental selection of test data. The whole procedure is repeated for test set sizes of 20 %, 50 %, and 80 %, to estimate the effect of an increasing amount of historical knowledge one might have obtained during p‐OWF analysis.

**Figure 7:**
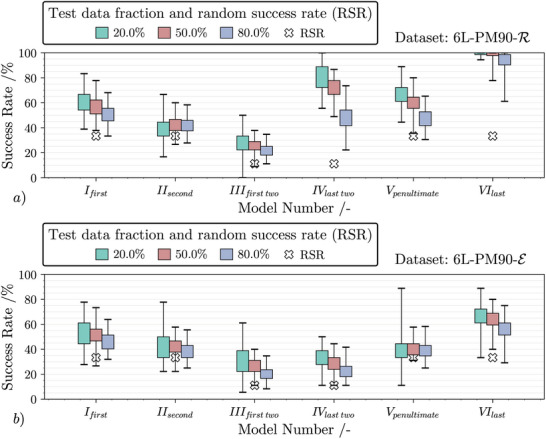
Box plot comparing model success rate of datasets 6L‐PM90‐R a) and 6L‐PM90‐E b) for three different sizes of test data after 100 iterations of randomized train test splits. Each model is shown separately. White crosses mark the average success rate when randomly guessing the corresponding pigment type(s).

The success rates for dataset 6L‐PM90‐R are visualized by box plots in **Figure** **7**a. As expected, the success rate scales with the size of the training data set. The more input‐output‐pairs of the p‐OWF are available, the easier it is for the DTC to predict the correct colors of each individual layer based on the transformed spectra. As already indicated in Figure 6, the reflected optical signal is strongly influenced by the uppermost pigment layers and hence, these layers are easier to predict. In other words, models IV, V and especially VI achieve success rates of over 70% in the upper quantile. Figure 7 also shows the random success rate (RSR) that is achieved by simply guessing the corresponding pigment type(s) randomly and therefore resembling the trial‐and‐error procedure during classical PoW. Every time there is a discrepancy between the RSR and the computer‐aided prediction, having models like these helps in solving the inverse problem. In this case, the models provide the pigment type of the last layer with high reliability, which reduces the number of possible permutations from 90 to 30. Figure 7 shows that the data‐driven models are not able to reliably predict the exact pigment types in the bottom‐most layers, although the average discrepancy to the RSR indicates a distinct advantage over pure trial‐and‐error. Keep in mind that the collision analysis showed that the bottom‐most layers still have a significant effect on the reflection spectra, which appears to be not predictable by the DTC models based on the provided data. Otherwise, nearly all samples would result in collisions, i.e. identical spectra that are only dependent on the top layer. This shows that printing a multilayer structure is essential for non‐invertibility.

The results for 6L‐PM90‐E are shown in Figure 7b. The results for 4L‐P81‐E are similar and given in Figure S6 (Supporting Information). In contrast to the reflection data, the average success rates are only 10−30% higher than the RSR throughout all layers. In other words, there is no clear difference between the prediction accuracy of the first and final pigment layers. From a physical standpoint, this observation is not surprising because signal detection occurs after the light has passed uniformly through each individual pigment layer. The overall low success rates are good in terms of non‐invertibility and indicate that predicting the exact sequence from a given spectrum is hard. However, it must be noted that an average collision rate of ≈3.5 % was obtained for this data set. Some falsely predicted sequences might therefore still yield the same, correct spectrum after printing and analysis, if they collide with the correct sequence.

### 2.4 Interim Conclusion on the Proposed Physical One‐Way Function

The optical analysis of pigment structures showed low experimental errors and can be considered **steady**. Although still small, the printing process produced larger experimental errors. Samples printed on paper were found to be more **reproducible** than samples printed on translucent foil indicating that the substrate plays an essential role. However, reproducibility must always be judged in conjunction with collision resistance, since we have to define and discretize what a “correct” answer is based on the experimental deviations. The reflection data set can be considered **collision resistant** with a collision probability of only 0.1 %, while the extinction data showed collision probabilities above three percent. Regarding **non‐invertibility**, a PCA revealed that predicting the overall pigment composition, i.e. “color,” is successful with a high probability. In the extinction setup, the PCA did not reveal any distinct patterns, while the reflection data can be grouped based on the pigment of the uppermost layer. This effect is also apparent when training data‐driven DTC models for the inverse problem: High prediction accuracy is obtained for the upper‐most layers, while predicting the pigments in the bottom‐most layers is still challenging and not drastically enhanced compared to trial‐and‐error. Although slight improvements were recorded, the trained models did not reliably reveal the printing sequence for the extinction setup.

Based on the gathered data and established boundary conditions for the security requirements, we state that the proposed p‐OWF is suitable for a PopW environment, although further investigations especially into the non‐invertibility are required. Complexity can be increased in various ways, like e.g. increasing the number of layers or including lighting parameters in the input space.

### 2.5 Conceptualization of a Proof of Physical Work Framework

Assuming that all security requirements are met, a proof of physical work system can be conceptualized to illustrate the possible application of the developed p‐OWF. This is done by envisioning a hypothetical cryptocurrency called “Colorcoin” that functions identically to Bitcoin but employs the proposed PopW framework. Note that the following mining procedure was not tested in a real‐world scenario. This chapter is aimed at providing context toward possible application scenarios for the investigated p‐OWF.

A standardized machine called “mining printer” processes digital RGB color sequences and produces print samples comparable to those described in this study. A total of four spatially separated light sources simultaneously illuminate the sample with adjustable light intensities between 20 % and 100 %. Each light source has a constant, unique and pre‐defined wavelength‐specific spectrum. A built‐in sensor then records an UV/vis reflection spectrum of the pigment structure. The device driver must be set up to print a consistent amount of cyan, magenta, yellow and black pigments within a layer. In other words, a color set within the RGB color space is converted into a physical print in the CMYK color space.

The mining procedure involves the addition of a new block to the Colorcoin blockchain and its decentralized validation. First, each miner creates a physical puzzle utilizing the (digital) cryptographic hash function SHA‐256. The procedure is deterministic and starts with a ledger of transactions that a miner wants to validate and add to the blockchain. It also includes the coinbase transaction, which awards the miner for his or her work and therefore, the resulting puzzle is personalized. As a mandatory security requirement, this block of transactions is extended with the so‐called blockHeader. This procedure is identical to the Bitcoin protocol (except the inclusion of a nonce) and a schematic representation is given in **Table** **2**. The header includes a 32 bit sized protocol number (blockVersion) a 256 bit sized cryptographic hash of the previous block (hashPrevBlock) a 256 bit sized commitment hash of the transaction list (hashMerkleRoot) a 32 bit sized difficulty (puzzleDiff) and same sized timestamp (puzzleStamp).

**Table 2:** Schematic structure of a Bitcoin block header used in this proposal.

Field	Value	Size
blockVersion	00000001	32 bits
hashPrevBlock	a93695 … a46c40	256 bits
hashMerkleRoot	d6c335 … 863928	256 bits
puzzleDiff	00000001	32 bits
timeStamp	6603d416	32 bits

A combination of this information is passed as input to the cryptographic hash function SHA256, which then provides *D* pairs of unique *Puzzle Hashes* used to construct the mining task. The factor *D* is defined by puzzleDiff in decimal notation. Both hashes are calculated with the expressions

(5) $$\begin{array}{cc} {\text{SHA256}}^{2D}\left(\mathrm{blockHeader}\right) & ={\mathrm{blockHash}}_{D} \end{array}$$

(6) $$\begin{array}{cc} \text{and}\;\text{SHA256}({\mathrm{blockHash}}_{D}) & ={\mathrm{targetHash}}_{D}\;. \end{array}$$

Here, dSHA256^2*D*^ stands for the 2*D*‐times application of SHA‐256 on the blockHeader to obtain blockHash_*D*_. One more application of SHA256 yields the targetHash_*D*_. The following steps of the mining routine are explained using the example *D* = 1 and must be repeated for higher difficulties, i.e. all *D* pairs of *Puzzle Hashes*. Similar to Bitcoin, the difficulty can be automatically adjusted by the network to ensure a specific amount of verified blocks per given time interval.

**Figure 8:**
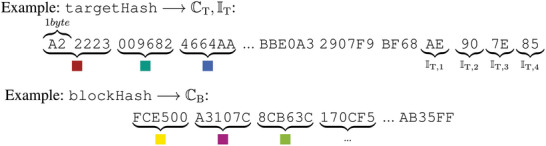
Schematic explanation of hash parsing and conversion into RGB colors saved in $C_{B}$ (block colors) / $C_{T}$ (target colors) and light intensity values for four lamps saved in tuple $I_{T}$.

There is an infinite amount of ways to convert both *Puzzle Hashes* into a set of RGB colors, as described by Seidemann and Jelich.^[^ ^23^ ^]^ For now, both the blockHash and targetHash are divided into 24‐bit‐sized chunks, each represented by six hexadecimal digits. Each chunk represents an RGB‐color in the standardized HTML format (#RRGGBB), with 2 hexadecimal digits (0‐255 in decimal) for each color red, green and blue. These first six colors are stored consecutively in the arrays $C_{B}$ (block colors from blockHash) and $C_{T}$ (target colors from targetHash). Additionally, the last four pairs of hexadecimal digits (bytes) from the targetHash are transformed into a tuple of four light‐intensity settings

(7) $$I_{T}=\left(\frac{I_{T,1}}{255},\frac{I_{T,2}}{255},\frac{I_{T,3}}{255},\frac{I_{T,4}}{255}\right)$$

scaled between 0 and 1 by the decimal value 255. Small numbers indicate that the light intensity of the lamp is close to its minimum value of 20 %, while one represents 100 % light intensity. The arbitrarily chosen minimum ensures that there will always be some light involved in the physical puzzle. A visual example of this procedure is shown in **Figure** **8**.

The digital pre‐processing steps are followed by the printing and analysis of the target sample. First, all six colors defined in $C_{T}$ are printed in array sequence on top of each other to create the target sample. The standardized lamps illuminate the generated pigment structures with light intensities set by $I_{T}$. This step produces an UV/vis reflection spectrum $S_{T}$ whose wavelength‐specific values represent the numerical targets of the puzzle. The Colorcoin miner's task is to now find a specific sequence $C_{B}^{*}$ by reordering of $C_{B}$ in addition to a light intensity setting $I_{B}$, so that the resulting spectrum $S_{B}$ after printing and analysis matches the spectrum $S_{T}$ in a predefined margin of error. Six layers offer a total of 6! = 720 unique color sequence combinations $C_{B}^{*}$, each of which can be combined with any of the 256^4^ illumination settings $I_{B}$, representing a vast input space. The non‐existence of a solution is highly unlikely, since different layers of the sample were shown to have more or less pronounced influence on the reflection spectrum and the four light sources produce unique spectra.

After finding a valid solution to all *D* individual puzzles, the non‐encrypted information of the correctly sorted print sequences $C_{B,i}^{*}$ and corresponding illumination configurations $I_{B,i}$ (*i* ∈ {1,…,D}) are appended to the blockHeader and broadcast to the network. To validate the authenticity of any solution broadcast across the network, any participant can simply print both the target and proposed solution sequence and compare the resulting spectra. Obviously, this requires all participants to use the same printing procedure, software and standardized hardware. Although less demanding, these prerequisites also apply during digital PoW, where the Bitcoin protocol has to be followed and standardized algorithms like SHA‐256 have to be used.

### 2.6 Other Considerations Beyond the Scope of This Work

1) **Different Output Spaces**: During this work, two entirely different outputs (extinction and reflection spectra) were generated from a single input (digital pigment sequence). In fact, performing different observations on the same physical pigment structure, like e.g. IR spectroscopy or X‐ray microtomography, produces many different output spaces. Although no direct link might exist from one specific output *o*_1_ back to the input *i*, it is impossible to guarantee this for all outputs. Assume that there is a analytical or data‐driven connection between output space *o*_1_ and *o*_2_, i.e. *o*_1_ → *o*_2_. Further assume that *o*_1_ is not invertible ($o_{1}\neg \to i$) but *o*_2_ is (*o*_2_ → *i*). In this case, the inverse problem based on *o*_1_ is easily solved by the path via *o*_2_ (*o*_1_ → *o*_2_ → *i*). Similarly, it is possible that although neither *o*_1_ nor *o*_2_ are invertible on their own, combining both outputs might indeed make the problem invertible (*o*_1_ + *o*_2_ → *i*). This means that for a p‐OWF to be non‐invertible, all relevant analytical tools must be taken into account.
2) **Pseudo‐Randomness of Cryptographic Hash Functions**: Changing the input to cryptographic hash functions like SHA‐256 in the slightest produces an entirely different output that – although deterministic – appears random. In the case of p‐OWF this is not the case: Changing the pigment in a specific layer will produce a different, but still similar optical response. This makes the inverse problem susceptible to optimization strategies, i.e. there might be more efficient ways of finding a corresponding input than trial‐and‐error. The investigated data‐driven models provide educated guesses on the correct pigment structure. From there, systematic variations of certain layers might quickly lead to the correct sequence. However, as long as there is still physical printing and optical analysis involved this merely reduces the required work but does not make it obsolete. In this case, the difficulty of the PopW can be adjusted accordingly.
3) **Anonymity of the Network Participants**: One of the major achievements of Bitcoin is that the network operates in a fully‐decentralized and anonymous way. In principle, the envisioned PopW does not change that, however, miners would require specific hardware and materials that are easier and more obvious to track than computer parts and electricity.
4) **Availability and Distribution of Materials**: All network participants need to be able to verify transactions reliably, which requires reliable access to materials, like electricity during classical PoW and pigments in the proposed PopW. This puts power into the hands of producers and distributors that might be misused to prioritize or even re‐write certain transactions via a 51% attack. The more specialized the materials, the higher is this risk.
5) **Environmental and Ethical Considerations**: The Discussion S1 (Supporting Information) provides an example calculation that illustrates the envisioned CO_2_ reduction for p‐OWFs in a simple way based on certain assumptions. Reality is more complex than that and is obviously tied to the details of the actual PopW system. Ecological benefits, but also ethical considerations, like where and under which conditions the required materials are produced need to be specifically investigated in an interconnected setup, where offer, demand, prices, etc. are simulated. Note that this might also mean that the PopW can be designed even more sustainable, e.g. when pigments and substrates can be recycled.
6) **Meaningfulness of the Work Performed**: In a digital PoW, the work effort consist of repeated evaluations of cryptographic hash functions and is more or less “useless” besides maintaining the blockchain. In a PopW however, this work is based on a physical puzzle i.e. dependencies in our world that are not understood entirely up to this point. Even if, in the long run, enough data is collected to actually invert the p‐OWF and collapse the PopW system, this inversion might be reused to serve a greater purpose. Scheduled changes of the PopW parameters, like e.g. new pigments each year, could help to maintain security.

## 3 Conclusion

The transition from purely digital proof of work concepts to those rooted in the physical world represents an entirely new and highly interdisciplinary field of research. The cornerstone of such proof of physical work (PopW) systems are physical one‐way functions (p‐OWF). In principle, they share the same security requirements as their digital counterparts, but certain aspects such as their inherent experimental errors have to be considered and demand adjustment. This work establishes boundary conditions for these requirements, however, precise standards and strategies for challenging them need to be defined in future work, to claim that a p‐OWF can be considered “safe.” The experimental investigations of the pigment‐based p‐OWF example show the general feasibility and provide guidelines on which properties are essential and how they can be assessed. Applying the exemplary p‐OWF, a PopW framework is conceptualized that is able to significantly reduce the CO_2_ emissions compared to traditional PoW‐based systems such as Bitcoin. The proposed p‐OWF and PopW should not be misunderstood as the only solution for this challenge: The aim is to promote interest and spark discussions in a wide range of disciplines, including cryptography, chemistry, physics, and engineering to identify other p‐OWF/PopW setups.

## 4 Experimental Section

### Sample Preparation and Spectroscopic Measurement

**Figure 9:**
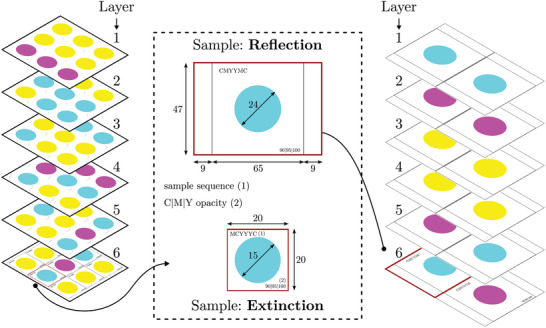
Visualization of the printing procedure using computer‐generated print pages with individual color layers for extinction (left) and reflection (right). All dimensions are in mm.

The p‐OWF procedure consisted of printing specific pigment sequences on a fixed position on a substrate with subsequent spectroscopic analysis of the resulting structure. Experiments were performed with the commercially available printer *Epson EcoTank ET‐4850* (SEIKO Epson CORPORATION, Suwa, Japan) using the stock printer ink with colors cyan (C), magenta (M) and yellow (Y). The varied print sample characteristics were the amount of used ink (pigment quantity) and their distribution (sequence) on the substrate. Since end‐users had restricted access to the core programming of the printer driver, an explicit conversion from digital RGB to physical mass of CMYK pigment was not possible. This problem was solved by only printing constant areas of pure pigment (C, M or Y). These color quanta have constant RGB values and size and the integral pigment amount was given by the discrete amount of printed layers. This procedure was presented schematically in **Figure** **9**. The coverage of pigment particles on the carrier surface marks an additional degree of freedom and was controlled via the color opacity of the printer, resulting in a more or less transparent sample.

In this work, two different spectroscopic measurements were performed on the pigment structures. The first one measures the quantity of reflected light in the visible range and the data set is denoted with R for reflectance. Samples were prepared using high‐quality paper (HQP) (tecno superior, Inapa Deutschland GmbH, Hamburg, Germany) as substrate. Following the printing of a single pure color layer with a predefined opacity setting, the sample was subjected to a 5 min drying process at a temperature of 30 °C, utilizing heat emitted by an infrared lamp. This routine was repeated until the desired amount of layers are printed on top of each other. The wavelength dependent reflection was measured between 361 and 800 nm and a spectral resolution of 1 nm with a UV/vis‐NIR spectrometer (*Cary 5000*, Agilent Technologies, Santa Clara CA, USA). Planarity of the samples was ensured by using the solid sample holder, specifically designed for filters, textiles, and other solid samples. The sample has two smaller areas to the left and right (see Figure 9) to allow for a correct alignment of the sample in the light beam. The illuminated sample area was 3.5 mm^2^ and located in the center of the sample. Considering a printed area of 452.4 mm^2^, small alignment errors during printing and analysis become irrelevant. During each measurement series, the absorption spectra are baseline‐corrected for the substrate and the electric background noise of the detector.

The second method was based on UV/vis extinction spectroscopy, quantifying the attenuation of light traversing the entire sample. The data set was denoted with E for extinction. For this, coated polyester foil (CPF) (Avery Inkjet 2502, Avery Zweckform GmbH, Oberlaindern, Germany) is used as substrate. The print settings and files were identical to those used in R. The acquisition of wavelength‐dependent extinction data between 380 and 800 nm and a spectral resolution of 0.423 nm is handled by a UV/vis spectrometer (*Flame S‐XR1*, Ocean Insight, Orlando, FL, USA) in combination with a deuterium‐halogen light source (*DH‐2000*, Ocean Insight, Orlando, FL, USA). Optical fibers guide the monochromatic light to a collimator lens. A custom sample holder centers the lens under the printed sample and ensures it's planarity. Again, only a fraction of the printed sample was measured to account for small alignment errors during printing and analysis. A second collimator lens was locked on top of the construction and gathers the attenuated light. As with reflection measurement, electric background noise of the detector and the influence of the substrate was accounted for by a reference measurements. Figure 9 provides an illustration of the samples and preparation procedure for both E and R with corresponding geometric dimensions. A photograph of the physical samples is provided in Figure S1 (Supporting Information). A comprehensive discussion of pigment application to the sample surface, supported by multiple imaging techniques and elemental spectroscopy, can be found in Discussion S2 (Supporting Information).

### Experimental Design

To analyze the influencing factors on both the E and R optical responses, a systematic variation of the pigment structure properties was required. As mentioned above, due to limited control over the printer driver, multiple layers of pigment quanta with adjustable opacity were printed in a defined sequence. All investigated samples were printed in triplicate and each sample was measured ten‐fold to assess reproducibility. The designation of each individual sample is as follows: First, the total number of layers *L* is defined. Next, each of the layers was set to one of the three available pigments, C, M or Y. Finally, the sample name was specified by the sequential combination of these three letters. For example, a sample named CCMMYY consists of two layers of pure cyan at the bottom, followed by two layers of pure magenta and two layers of pure yellow on top. In general, both the printing sequence and the amount of pigment per sample can be quantified by permutations found in combinatorics theory. The total scope of possible variation when *N* unique colors are printed on *L* layers is defined by the number of permutations with repetition

(8) $$P=N^{L}$$

Since this study on security requirements aims to isolate the factor color sequence in particular, the total set of permutations can be reduced by keeping the total number of printed layers of each pigment constant. This can be achieved by so‐called “multiset permutations.” The possible number of permutations in a multiset M is calculated with the expression

(9) $$P_{M}=L!{\left(\prod_{k=1}^{K}m_{k}!\right)}^{-1}$$

where *m*_*k*_ are so‐called multiplicities of each individual object in the set. The numerator is the factorial of the total object count. Here, this is the total amount of printed layers *L*. An example would be all anagrams of the above‐mentioned sample CCMMYY. The distinct multiplicities of cyan, magenta and yellow are defined by the number of occurrences in multiset M resulting in *m*_1_ = *m*_2_ = *m*_3_ = 2 and a layer size of *L* = 6.

Two permutation sets were investigated in this study. The first one, named 4L‐P81‐E, consist of samples with four layers of *N* = 3 base colors. All possible permutations were printed and hence, Equation (8) yields the total number of permutations of 81. The flag ‐E indicates, that only extinction was measured with this dataset. This dataset was used to investigate the color perception of the extinction spectrum. In other words, printing only four layers allows the human eye to still distinguish between samples. The second collection of print configurations focuses on the effects of color sequence on both reflection and extinction. For this, the multisets 6L‐PM90‐E and 6L‐PM90‐R of permutation sample CCMMYY were used. According to Equation (9), 90 individual prints are needed for a full‐factorial design. A deliberate decision was made in favor of only one multiset, as all permutations with repetition would have required the manufacturing of 3^6^ = 729 samples and their respective triplicates, which was not feasible in the scope of this work. However, the successive enlargement of the permutation database and their respective spectra is planned for future research.

**Figure 10:**
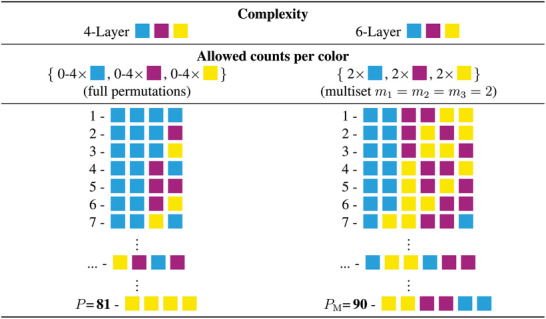
Overview of samples included in permutation set 4L‐P81‐E, 6L‐PM90‐E, 6L‐PM90‐R. Color sequence (print order) is depicted from left to right.

An illustration of nine individual print permutations for each set is depicted in **Figure** **10**. From this full‐factorial design of experiments (DOE), a python script generates printable PDF pages. All final .pdf files and the generator .py file are provided as File S1 (Supporting Information). During sample generation, the respective substrate is reinserted according to the number of layers that need to be printed. A full overview of all samples and their respective designations is listed in Tables S1–S3 (Supporting Information). A visualization of the printing procedure is given in Figure 9, while Figure S1 (Supporting Information) shows photographs of the physical samples.

## Conflict of Interest

Frank Kleine Jäger, Lothar Seidemann and Holger Jelich are currently employed by the funding organization BASF and involved in certain related patents. All other authors affiliated with KIT declare no financial or non‐financial competing interests and are not associated with any of the patents. The experimental investigations and data analysis were performed at KIT without any governance or control of BASF and associated authors. Hence, the conducted research is unbiased by financial interest.

## Author Contributions

M.W., L.S., H.J., F.K.J., J.M.Q., A.C., H.N., and F.R. contributed to conceptualization; M.W., C.P., S.P., and F.R. were responsible for methodology; M.W., C.P., S.P., and F.R. conducted formal analysis; investigation was carried out by M.W., C.P., and F.R.; data curation was performed by M.W.; writing of the original draft was done by M.W. and F.R.; writing (review and editing) involved M.W., L.S., H.J., F.K.J., J.M.Q., A.C., H.N., and F.R.; visualization was handled by M.W.; supervision was provided by F.R.; and funding acquisition was undertaken by L.S., H.J., J.M.Q., A.C., H.N., and F.R.

## Supporting information

Supporting Information

## Data Availability

The data that support the findings of this study are openly available at [https://doi.org/10.35097/PmiZWVEHnJcxNEHH] and the Python program used to generate the printed pages available at [https://doi.org/10.35097/MYkkeSPeePTDkkCY], reference number [1000172109].
