# Supplementary material for: Physical One‐Way Functions for Decentralized Consensus Via Proof of Physical Work
Source: Adv Sci (Weinh). 2024 Dec 16;12(5):2409386. doi: 10.1002/advs.202409386 (PMC11791968; doi:10.1002/advs.202409386)
Supplement: Supplementary file 1 — Supporting Information [file ADVS-12-2409386-s001.pdf]

ADVANCED SCIENCE
Open Access

Supporting Information

for *Adv. Sci.*, DOI 10.1002/advs.202409386

Physical One-Way Functions for Decentralized Consensus Via Proof of Physical Work

*Marvin Winkler, Catharina Peither, Simon Petrick, Lothar Seidemann, Holger Jelich, Frank Kleine Jäger, Jörn Müller-Quade, Alexander Colsmann, Hermann Nirschl and Frank Rhein**

Supplementary materials for

Physical one-way functions for decentralized consensus via proof of physical work

Marvin Winkler,[1,2] Catharina Peither,[1,2] Simon Petrick,[1,3,4]
Lothar Seidemann,[5] Holger Jelich,[5], Frank Kleine Jäger,[5]
Jörn Müller-Quade,[1,6] Alexander Colsmann,[1,3,4]
Hermann Nirschl,[1,2,3] Frank Rhein,[1,2,3*]

[1]Karlsruhe Institute of Technology (KIT),
[2]Institute of Mechanical Process Engineering and Mechanics (MVM),
Strasse am Forum 8, 76131 Karlsruhe, Germany
[3]Material Research Center for Energy Systems (MZE),
Strasse am Forum 7, 76131 Karlsruhe, Germany
[4]Light Technology Institute (LTI),
Engesserstrasse 13, 76131 Karlsruhe, Germany
[5]BASF SE, Carl-Bosch-Strasse 38, 67056 Ludwigshafen/Rhein, Germany
[6]Institute of Information Security and Dependability (KASTEL),
Am Fasanengarten 5, 76131 Karlsruhe, Germany

*To whom correspondence should be addressed; E-mail: frank.rhein@kit.edu.

This PDF file includes:

- Supplementary Discussion S1
- Supplementary Figure S1
- Supplementary Discussion S2
- Supplementary Figure S2
- Supplementary Figure S3
- Supplementary Figure S4
- Supplementary Figure S5
- Supplementary Figure S6
- Supplementary File S1
- Supplementary Tables S1, S2 and S3

Supplementary Discussion S1: Exemplary CO_2 calculation for the proposed setup

Stating that p-OWFs can contribute to reduce the carbon footprint of a cryptocurrency like Bitcoin is counter-intuitive at first. The fundamental assumption is that miners will only mine if it is profitable, i.e. that the average mining reward is higher than the invested resources in US-Dollars (USD). As of today (03/18/2024), the current reward for mining a block is 6.25 Bitcoin (BTC), which is roughly equivalent to 424,000 USD (c.f. `https://coinmarketcap.com/currencies/bitcoin/`). Taking into account the regional electricity prices and mining power distribution, the average bitcoin electricity price based on historical data (2018) was estimated at 0.091 USD/kWh (*1*). Thus, on average, the economical upper limit for the electricity consumption of any given block is 4.7 GWh. The CO_2 footprint based on the regional mining power distribution and respective average power mix was estimated at 557.76 g CO_2/kWh in 2021 (*2*), resulting in an estimated carbon emission of 2601 t CO_2 per mined block. The network is designed to adjust the difficulty of the inverse problem so that on average a block is mined every 10 min. This would result in a yearly CO_2 emission of 137.6 Mt/year, which is in the same order of magnitude as estimations from 2021 (*2*).

The proposed setup is based on and limited by printing of pigment particles originating from ink fluids. Eurocolour e.V., the umbrella organization for the manufacturers of pigments in Europe, lists the carbon footprint of multi-stage synthesized organic pigments at 24 kg CO_2/kg Pigment (*3*). It is impossible to quantify the precise pigment cost at this stage, as many factors such as quality and ink formulation have to be taken into account. The following calculation represents a worst-case estimate and assumes, for example, minimal dilution of the ink during formulation as well as the usage of expensive pigments prior to any marked adjustments by an elevated demand. For reference, a commercially available ink (Epson 113 EcoTank) costs around 10 USD per 70 mL and contains 5-30 % pigment (*4*), which yields a pigment price (post-formulation) of at least 476 USD/kg. An additional source lists the pigment fraction in ink fluids even lower at

2% to 5% (*5*). Accounting for large demand and therefore assuming a price of only 40 USD/kg in the final formulation, mining a block is only economically viable, when less than 10.6 t of pigment are being used. This results in a carbon footprint of 254.6 t CO_2 per block, which corresponds to a 90 % CO_2 reduction. Eurocolour e.V. also lists inorganic and metallic pigments whose carbon footprint per kg is significantly lower. Adjusting the calculation on the basis of these substances, with a higher dilutions, could would yield a CO_2 reduction of over 99%.

Furthermore, it can be argued that the calculations neglect the cost of the substrate for the pigment structure and potential recycling strategies. Both will significantly influence the carbon footprint calculations in one way or the other. Methods have already been conceptualized to circumvent these negative aspects, as suggested in patent (*6*). Here, the mining printer is based on the laser printing technology and contains a so-called transfer belt. Here, pigment suspensions are used to create the desired particle structures. Afterwards, they are directly on this machine part, so that no carrier material is required. After optical analysis, the transfer belt can be cleaned and only the pigments themselves enter the recycling process.

The calculations above are meant to prove the general point that a significant carbon footprint reduction is indeed possible without putting too much emphasis on the specific figures. Obviously, reality is multi-faceted and cannot be foreseen just now, as e.g. pigment price will be strongly correlated to the success of a PopW-based cryptocurrency and its currently unspecified architecture. However, the general argument holds that a p-OWF is limited by physical resources and time, both of which might require less CO_2 per USD of economic value than electricity. The time aspect is particularly interesting in this context, as it is colloquially money, i.e. expensive, but does not produce carbon emissions per se.

Supplementary Figure S1: Photograph of two exemplary samples.

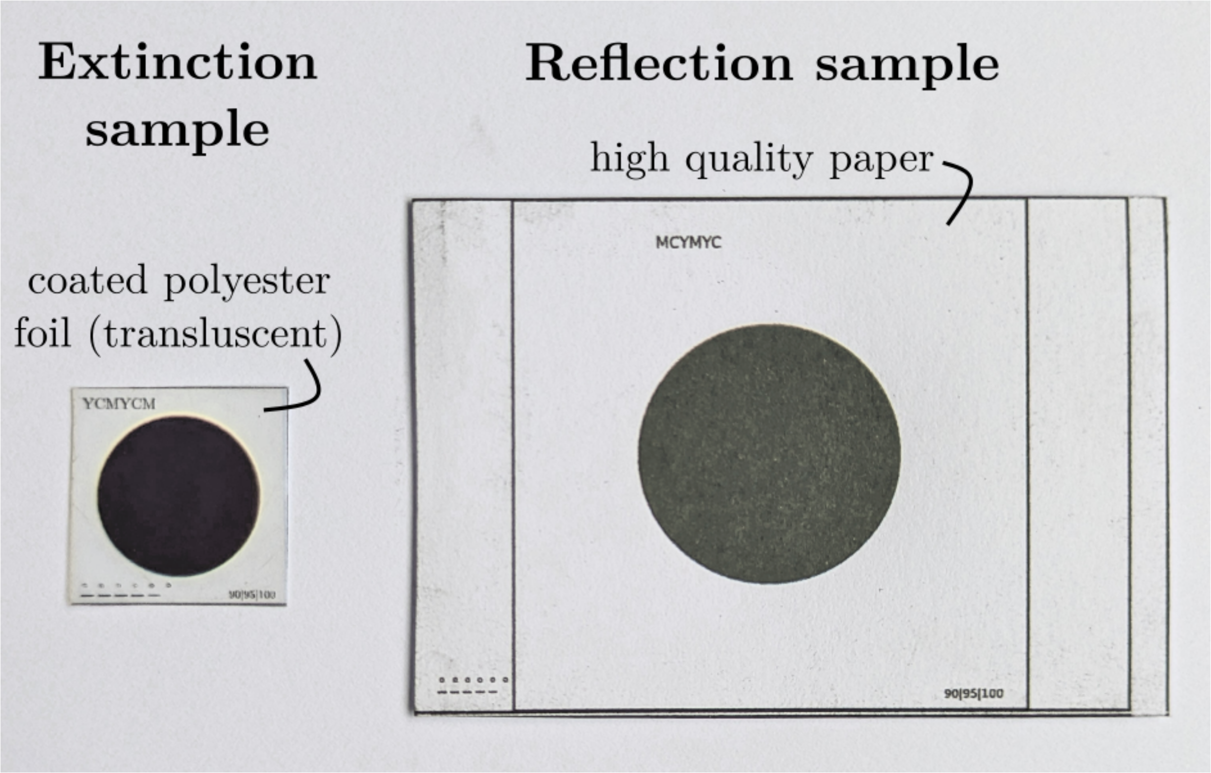

Figure S1: Photograph of two exemplary samples for extinction (left) and reflection (right).

Supplementary Discussion S2: Detailed description of sample characteristics.

This section elaborates on the physical and chemical characteristics of each sample and materials used to create a colored print. First, both high quality paper (HQP) and the coated polyester foil (CPF) in combination with the used molecular dyes of the ink-jet printer are described more thoroughly. Both substrates were analyzed by scanning electron microscopy (SEM) and energy discursive X-ray spectrometry (EDX). Samples of two prints with color sequence CMYCMY were prepared as shown in Figure S3e. Figure S4 shows EDX spektra of the pure HQP and CPF material. The main peaks are associated with calcium (Ca), carbon (C) and oxygen (O) for the HQP and aluminum (Al), oxygen (O) and carbon (C) for the CPF. This suggests that both carriers are coated with $CaCO_3$ and Al_2O_3 respectively.

Regarding white print paper, porous coatings made of calcium carbonate are a common feature to increase the quality of prints. The reason for this is that the mineral coating affects the interaction of light with both the paper itself and the applied molecular dyes and pigments, influencing properties such as whiteness, shading, and opacity. The light scattering of the coated layer can be optimized by adjusting the distribution, mean particle size and shape of $CaCO_3$ particles on the paper surface (*7*). Regarding the HQP used in this study, small $CaCO_3$ particle clusters are visible in Figure S3a and S3b on top and inside of the paper fibers. Additionally, since ink-jet ink predominantly consists of water, porous coatings assist in water absorbance and inhibit the running of ink on the substrate. Another common material for surface coatings in printing of paper and films is aluminum oxide (*5*), which is applied during production of the CPF introduced in the manuscript. Both SEM images on the right side of Figure S3 show a porous structure on top of the foil. The coating offers the same advantages as the $CaCO_3$ clusters when liquid dye is sprayed onto the carrier surface during printing.

A significant question that must be addressed is how the molecular dye ink, applied in multiple

print and drying sequences as illustrated in Figure ??, interacts with the substrate surfaces to produce the final colored sample. A plausible understanding of the final product can be seen in Figure S2. Here, three light scanning microscope (LSM) images of three different samples are displayed. The main manuscript examined the rationale behind the subtle yet noticeable variations in color observed between the three individual samples. It was demonstrated that the sequence in which the dye molecules are sprayed on the carrier surface affects the UV/vis spectrum and, consequently, the color perception. Upon initial examination of Figure S3, however, it appears that the surface structure is identical regardless of whether the dye ink was applied. This means that the dye molecules are too small to be detected with the SEM image analysis. Furthermore, it is likely that the liquid dye molecules are transported to the fine pores of the coatings where they crystallize to the solid state. Successive printing and drying of the ink results in a layered structure of nanoparticles inside the porous materials.

In simplified terms, the scattering of light by small particles depends on the particle size of a volume-equivalent sphere. Additionally, the entire collection of UV/vis extinction bands is the sum of scattered and absorbed light intensities by illuminated particles. Particles below 380 nm have a lower scattering cross section and contribute to the absorbance of light predominantly because of their molecular structure (*4*), (*8*). Following the cited fundamentals it can be concluded that the incident light is scattered and absorbed by nanoscale pigment clusters inside the coating of the HQP and CPF. In conclusion, layers of crystallized dye molecules are formed inside the small pores that enable the application concept of a p-OWF described in the main manuscript.

Supplementary Figure S2: Laser Scanning Microscope images of selected print samples.

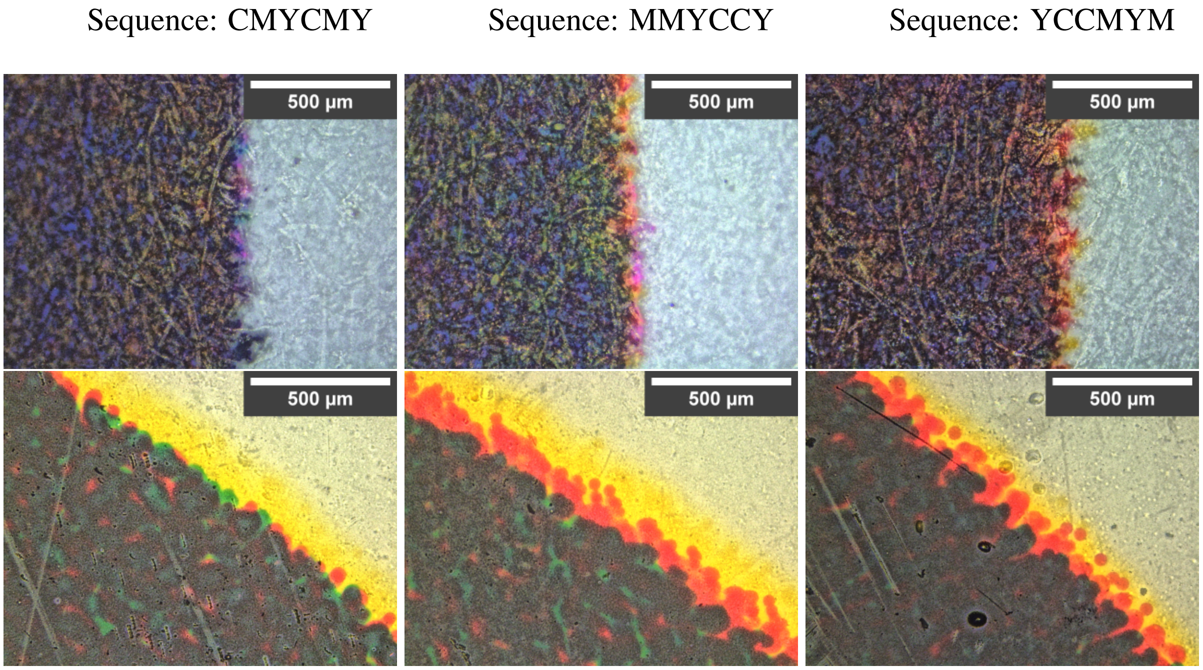

Figure S2: Images of print samples acquired with laser scanning microscope (LSM). Each column displays a sample used in reflection (paper substrate, top row) and extinction (foil substrate, bottom row) analysis of three different primary color sequences. According to these LSM images, particle structures displayed in the bottom row share nearly the same perceptible color whereas particle structures printed on paper (top row) show a noticeable difference in coloration.

Supplementary Figure S3: Substrate surface analysis by SEM.

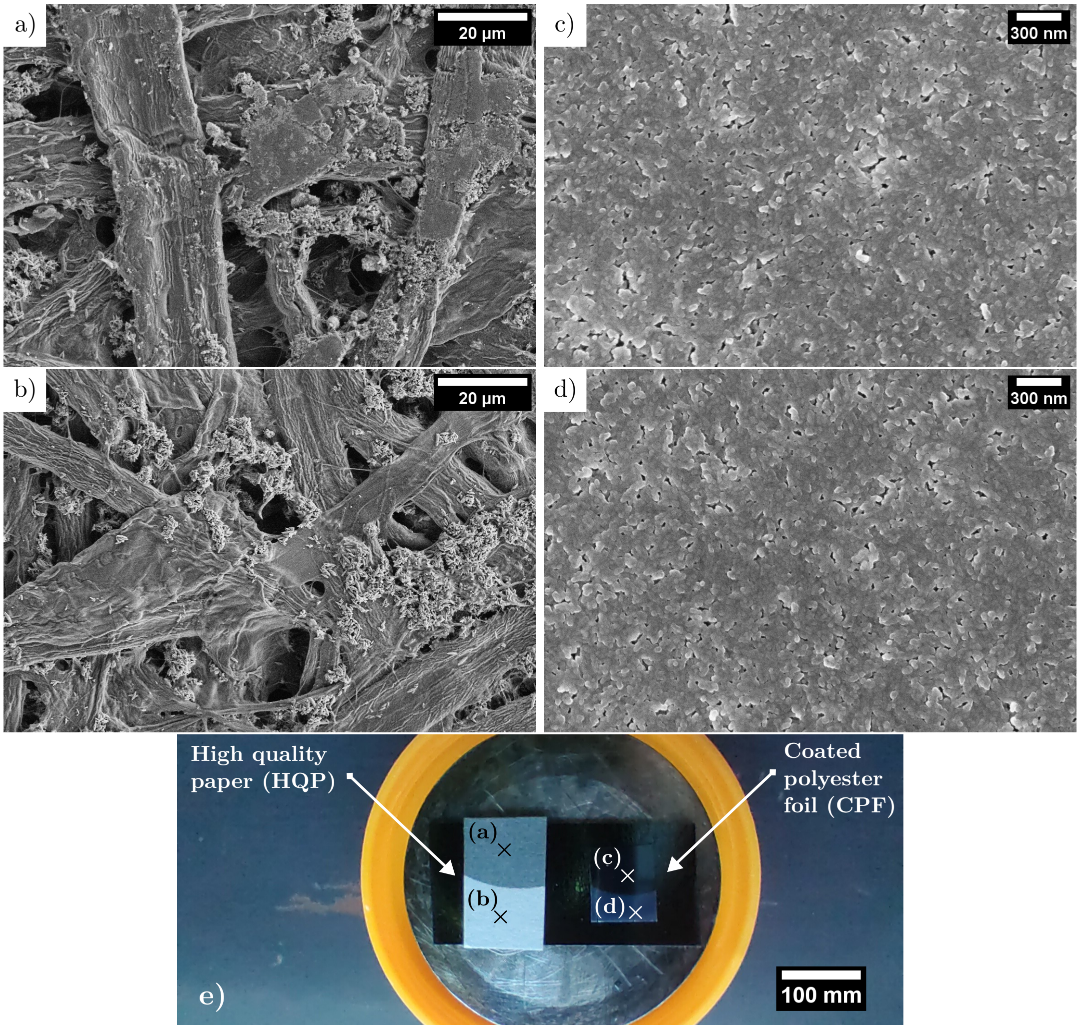

Figure S3: Scanning electron microscopy (SEM) images of two samples with six pigment layers (CMYCMY) printed on high quality paper (HQP, left) and coated polyester foil (CPF, right). Images a) and c) show pure substrate surface structures. Images b) and d) show the same structures with six layers of dye ink printed on top. The final image e) depicts both samples with no magnification. Labeled crosses mark the spots where each SEM image was acquired.

Supplementary Figure S4: Preliminary substrate analysis by EDX spectroscopy.

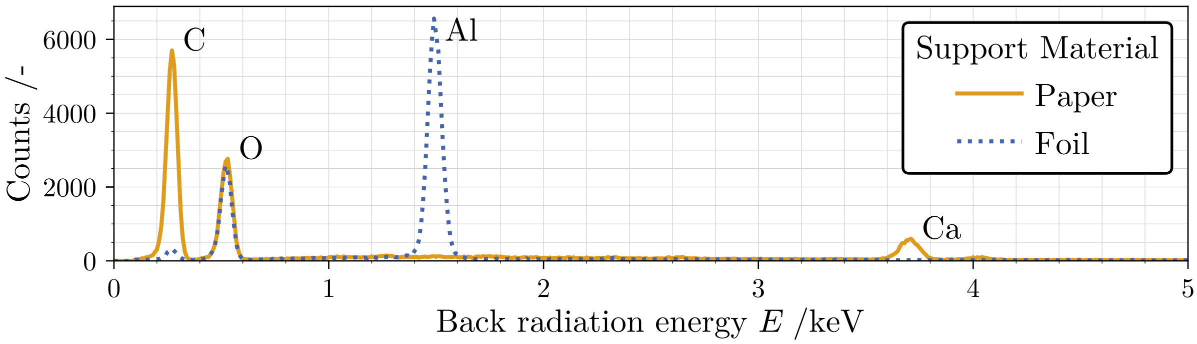

Figure S4: Energy dispersive X-ray spectrometry (EDX) spectrum of high quality paper (HQP) and coated polyester foil (CPF) used as substrate for printing. Elemental composition is indicated qualitatively by four labeled peaks.

Supplementary Figure S5: Standard deviations of data set 6L-PM90-$\mathcal{R}$.

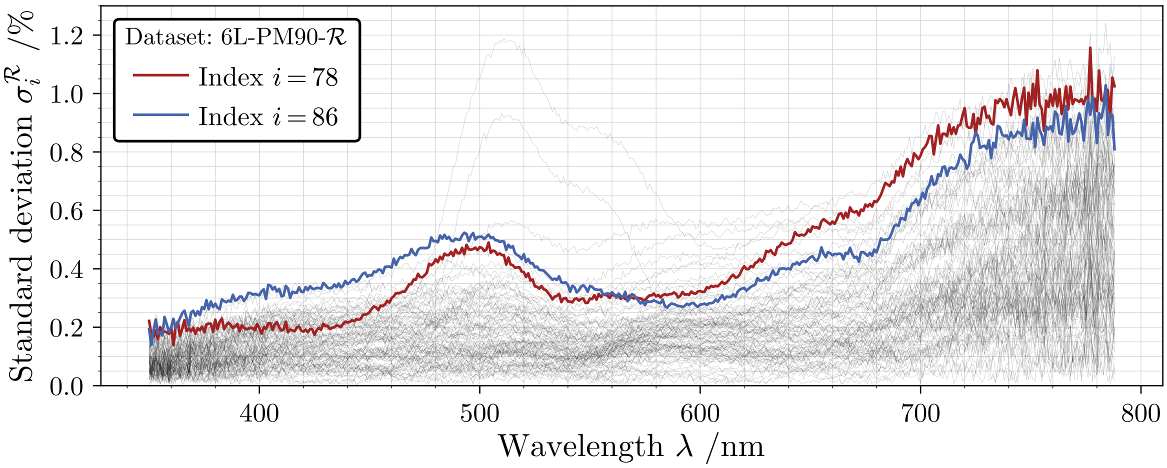

Figure S5: Calculated standard deviation σ_i of optical signals at multiple wavelengths for sample set 6L-PM90-$\mathcal{R}$. Samples with index 78 and 86 are highlighted in a red and blue color.

Supplementary Figure S6: Results of data-driven inversion for 4L-P81-$\mathcal{E}$ data set.

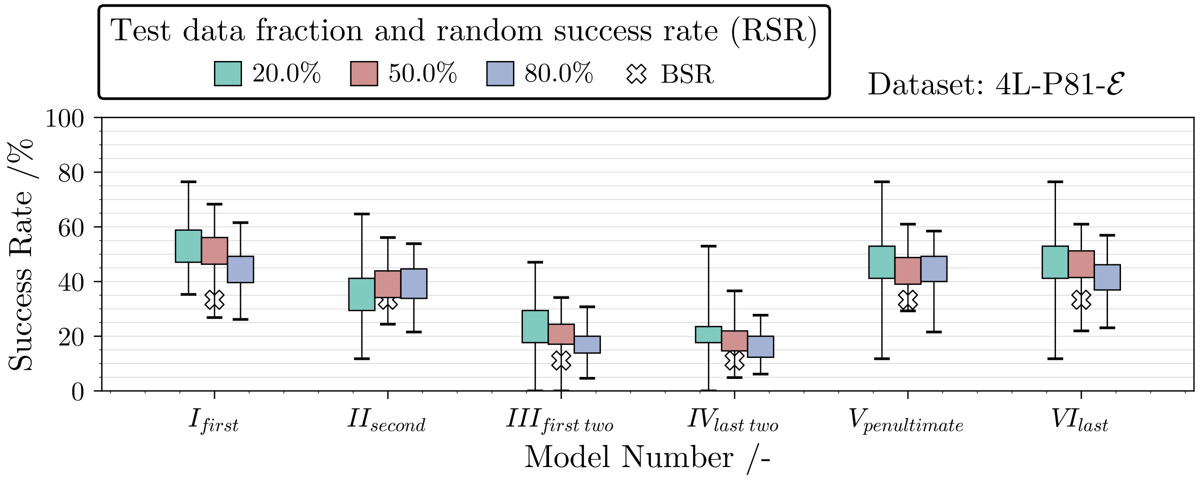

Figure S6: Box plot comparing model success rate for three different sizes of test data after 100 iterations of randomized train test splits. Each model is shown separately. White crosses mark the average success rate when guessing a print setting. The prediction whisker limits represent the minimum and maximum success rate achieved by each model.

Supplementary File S1: Printer files and Python generator script

The Python program used to generate the printed pages is available under the DOI: 10.35097/MYkke-SPeePTDkkCY.

Supplementary Tables S1–S3: Run tables of all experimental samples with mean standard deviations and values for principal components

The raw data used in this study are openly available under the DOI: 10.35097/PmiZWVEHn-JcxNEHH.

References

1. Küfeoğlu, S. & Özkuran, M. Bitcoin mining: A global review of energy and power demand. *Energy Research & Social Science* **58**, 101273 (2019).

2. de Vries, A., Gallersdörfer, U., Klaaßen, L. & Stoll, C. Revisiting bitcoin's carbon footprint. *Joule* **6**, 498–502 (2022).

3. Eurocolour e. V. Eurocolour flyer on product carbon footprint. `https://eurocolour.org/media/eurocolour_flyer_carbon_footprint_05.2022.pdf` (2022). Accessed: 03-18-2024.

4. Kipphan, H. *Handbook of print media: technologies and production methods* (Springer Science & Business Media, 2001).

5. Lee, H., Joyce, M., Fleming, P. & Cawthorne, J. Influence of silica and alumina oxide on coating structure and print quality of ink-jet papers. *Tappi Journal* **4**, 11–16 (2005).

6. Seidemann, L., Petersen, H., Rainer, F., Oliver, P. & Jelich, H. K. P. New method for pseudo-random number generation for information encryption (international patent no. WO/2021/001147 A1).

7. Kumar, N., Bhardwaj, N. K. & Chakrabarti, S. K. Influence of particle size distribution of calcium carbonate pigments on coated paper whiteness. *Journal of Coatings Technology and Research* **8**, 613–618 (2011).

8. Bohren, C. F. & Huffman, D. R. *Absorption and scattering of light by small particles* (Wiley-VCH, Weinheim, 2004). URL `http://swbplus.bsz-bw.de/bsz117808598cov.htm`.

Table S1: List of labels, mean standard deviation and principal components (PCs) for every sequence in dataset 4L-P81-$\mathcal{E}$.

Sample index i	Sequence	Mean STD $\bar{\sigma}_i^E$ /-	PC1 /-	PC2 /-	PC3 /-	PC4 /-	PC5 /-
0	CCCC	0.025	1.947	0.388	4.373	-0.007	0.859
1	CCCM	0.028	1.537	-0.476	1.856	0.723	-0.503
2	CCCY	0.02	1.494	0.889	1.197	-1.834	0.617
3	CCMC	0.023	1.546	-0.417	2.08	0.619	-1.035
4	CCMM	0.016	0.86	-0.883	-0.039	2.172	0.548
5	CCMY	0.014	0.876	0.027	-0.637	-0.336	0.075
6	CCYC	0.016	1.509	0.888	1.482	-1.787	0.377
7	CCYM	0.014	0.85	-0.055	-0.6	-0.33	-0.017
8	CCYY	0.022	0.851	1.327	-0.799	-0.568	1.523
9	CMCC	0.02	1.52	-0.541	1.948	0.719	-1.379
10	CMCM	0.032	0.808	-1.057	-0.064	1.844	0.103
11	CMCY	0.023	0.813	-0.125	-0.63	-0.332	-0.4
12	CMMC	0.033	0.727	-1.083	0.119	1.733	0.153
13	CMMM	0.025	-0.193	-1.46	-0.342	1.352	2.023
14	CMMY	0.023	-0.249	-0.677	-0.596	-0.699	0.443
15	CMYC	0.03	0.823	-0.172	-0.593	-0.441	-0.568
16	CMYM	0.025	-0.215	-0.875	-0.718	-0.621	-0.236
17	CMYY	0.023	-0.265	0.405	-0.73	-0.245	0.179
18	CYCC	0.022	1.49	0.841	1.424	-1.822	0.284
19	CYCM	0.02	0.814	-0.12	-0.6	-0.303	-0.175
20	CYCY	0.02	0.786	1.371	-0.715	-0.476	1.464
21	CYMC	0.027	0.763	-0.159	-0.506	-0.403	-0.602
22	CYMM	0.022	-0.242	-0.869	-0.631	-0.665	-0.405
23	CYMY	0.038	-0.273	0.373	-0.626	-0.323	-0.509
24	CYYC	0.027	0.819	1.318	-0.73	-0.677	1.108
25	CYYM	0.03	-0.269	0.292	-0.66	-0.235	-0.919
26	CYYY	0.035	-0.274	2.095	-0.671	1.039	0.688
27	MCCC	0.022	1.496	-0.517	2.012	0.902	-1.288
28	MCCM	0.027	0.773	-1.088	-0.053	2.024	0.122
29	MCCY	0.027	0.816	-0.106	-0.593	-0.303	-0.42
30	MCMC	0.025	0.735	-1.209	-0.017	2.064	-0.345
31	MCMM	0.027	-0.206	-1.545	-0.342	1.442	1.808
32	MCMY	0.026	-0.237	-0.851	-0.689	-0.505	-0.367
33	MCYC	0.017	0.735	-0.026	-0.473	-0.223	-0.197
34	MCYM	0.024	-0.244	-0.792	-0.7	-0.393	-0.376
35	MCYY	0.023	-0.262	0.46	-0.74	-0.166	-0.302
36	MMCC	0.028	0.765	-1.23	-0.05	2.024	-0.463
37	MMCM	0.031	-0.189	-1.537	-0.391	1.568	1.603
38	MMCY	0.036	-0.21	-0.817	-0.727	-0.54	-0.642
39	MMMC	0.031	-0.186	-1.558	-0.311	1.323	1.342
40	MMMM	0.025	-1.517	-1.378	1.072	-0.191	4.526
41	MMMY	0.027	-1.539	-0.971	0.874	-1.398	1.174
42	MMYC	0.021	-0.196	-0.831	-0.754	-0.513	-0.807
43	MMYM	0.029	-1.549	-0.901	0.875	-1.237	1.296
44	MMYY	0.015	-1.591	-0.035	0.903	-0.436	-0.459

Table S1: cont.

Sample index i	Sequence	Mean STD $\bar{\sigma}_i^E$ /-	PC1 /-	PC2 /-	PC3 /-	PC4 /-	PC5 /-
45	MYCC	0.026	0.795	-0.111	-0.612	-0.287	-0.442
46	MYCM	0.015	-0.219	-0.821	-0.729	-0.408	-0.555
47	MYCY	0.025	-0.248	0.487	-0.775	-0.096	-0.355
48	MYMC	0.018	-0.227	-0.812	-0.744	-0.351	-0.53
49	MYMM	0.025	-1.549	-0.956	0.88	-1.317	1.307
50	MYMY	0.014	-1.584	-0.182	0.909	-0.662	-0.792
51	MYYC	0.027	-0.244	0.484	-0.791	-0.082	-0.343
52	MYYM	0.029	-1.591	-0.135	0.921	-0.583	-0.673
53	MYYY	0.034	-1.637	1.275	1.019	1.019	-0.866
54	YCCC	0.032	1.485	0.8	1.419	-1.747	0.273
55	YCCM	0.03	0.801	-0.292	-0.626	-0.443	-0.968
56	YCCY	0.022	0.787	1.353	-0.739	-0.46	1.389
57	YCMC	0.031	0.779	-0.265	-0.626	-0.345	-0.956
58	YCMM	0.022	-0.238	-0.94	-0.704	-0.46	-0.599
59	YCMY	0.021	-0.266	0.274	-0.705	-0.279	-0.872
60	YCYC	0.022	0.79	1.36	-0.708	-0.55	1.275
61	YCYM	0.03	-0.266	0.286	-0.691	-0.217	-0.957
62	YCYY	0.024	-0.282	2.116	-0.679	1.169	0.817
63	YMCC	0.025	0.774	-0.184	-0.567	-0.304	-0.719
64	YMCM	0.027	-0.222	-0.95	-0.731	-0.46	-0.824
65	YMCY	0.024	-0.249	0.378	-0.805	-0.177	-0.605
66	YMMC	0.02	-0.228	-0.856	-0.678	-0.4	-0.746
67	YMMM	0.029	-1.537	-0.994	0.873	-1.433	1.185
68	YMMY	0.034	-1.576	-0.236	0.913	-0.764	-0.882
69	YMYC	0.035	-0.208	0.36	-0.808	-0.277	-0.85
70	YMYM	0.02	-1.624	-0.222	0.951	-0.546	-0.624
71	YMYY	0.034	-1.655	1.188	1.023	1.037	-0.975
72	YYCC	0.024	0.797	1.377	-0.744	-0.362	1.622
73	YYCM	0.02	-0.249	0.282	-0.801	-0.163	-0.834
74	YYCY	0.03	-0.264	2.121	-0.755	1.237	0.935
75	YYMC	0.023	-0.231	0.376	-0.845	-0.003	-0.482
76	YYMM	0.026	-1.611	-0.215	0.943	-0.57	-0.635
77	YYMY	0.026	-1.649	1.149	1.047	0.928	-1.141
78	YYYC	0.024	-0.261	2.125	-0.726	1.112	0.807
79	YYYM	0.024	-1.647	1.124	1.044	0.887	-1.206
80	YYYY	0.027	-1.692	2.941	1.158	2.818	-0.078

Table S2: List of labels, mean standard deviation and principal components (PCs) for every sequence in dataset 6L-PM90-$\mathcal{E}$.

Sample index i	Sequence	Mean STD $\bar{\sigma}_i^E$ /-	PC1 /-	PC2 /-	PC3 /-	PC4 /-	PC5 /-
0	CCMMYY	0.015	-1.688	0.591	3.064	3.382	3.225
1	CCMYMY	0.017	-1.757	0.546	0.871	1.87	2.014
2	CCMYYM	0.024	-1.043	0.285	0.72	1.381	1.526
3	CCYMMY	0.013	-0.459	0.96	0.404	1.393	1.604
4	CCYMYM	0.016	-0.464	0.663	-0.821	0.676	1.366
5	CCYYMM	0.016	-0.612	0.725	-1.428	0.02	-0.079
6	CMCMYY	0.011	-0.073	2.43	1.312	1.339	1.798
7	CMCYMY	0.028	0.44	2.035	-1.269	-0.129	0.872
8	CMCYYM	0.034	0.934	2.229	-1.675	-0.953	0.556
9	CMMCYY	0.016	0.489	1.957	-0.328	-0.03	0.75
10	CMMYCY	0.022	1.332	-0.277	-0.008	1.268	0.75
11	CMMYYC	0.025	2.182	-1.882	0.631	0.882	1.64
12	CMYCMY	0.013	0.034	0.912	0.049	0.387	0.452
13	CMYCYM	0.017	0.195	0.365	-0.381	-0.3	-0.466
14	CMYMCY	0.025	-1.514	-1.506	-0.6	1.398	0.045
15	CMYMYC	0.016	-0.955	-1.605	-0.189	0.037	0.955
16	CMYYCM	0.021	-1.02	-0.523	-0.691	0.3	-0.57
17	CMYYMC	0.019	-0.233	-0.578	-0.187	-0.664	0.456
18	CYCMMY	0.017	-0.26	0.288	-0.824	1.686	0.31
19	CYCMYM	0.015	0.23	0.838	-1.465	0.149	0.71
20	CYCYMM	0.039	-0.348	0.265	-0.526	-1.93	2.994
21	CYMCMY	0.01	1.197	-0.201	-1.006	1.322	0.195
22	CYMCYM	0.013	1.034	0.627	-0.854	0.094	0.211
23	CYMMCY	0.021	1.331	-0.392	-0.857	0.897	0.03
24	CYMMYC	0.023	1.866	-1.402	0.039	0.325	1.102
25	CYMYCM	0.03	0.76	-0.841	-0.204	0.373	-0.608
26	CYMYMC	0.026	1.46	-2.232	1.3	-0.184	0.009
27	CYYCMM	0.018	0.336	0.213	-1.352	-0.518	-0.117
28	CYYMCM	0.018	-1.795	-1.818	-0.898	0.454	-0.811
29	CYYMMC	0.02	-1.643	-1.849	-0.086	-0.889	0.333
30	MCCMYY	0.018	-1.45	1.027	0.366	1.659	-0.55
31	MCCYMY	0.017	-1.2	0.735	-0.397	0.884	-0.628
32	MCCYYM	0.02	-0.526	0.954	-0.384	0.129	-0.4
33	MCMCYY	0.012	0.693	-0.029	-0.191	1.346	-0.809
34	MCMYCY	0.01	0.33	0.201	-0.56	1.101	-0.971
35	MCMYYC	0.024	0.481	-0.036	0.06	-0.55	0.9
36	MCYCMY	0.02	1.069	-0.75	-0.398	1.1	-0.058
37	MCYCYM	0.02	1.886	-0.536	0.374	1.173	-0.786
38	MCYMCY	0.018	1.377	-0.202	-0.685	0.811	-0.324
39	MCYMYC	0.023	0.308	0.043	0.342	-0.74	0.989
40	MCYYCM	0.03	0.769	-0.71	1.192	0.839	-1.023
41	MCYYMC	0.013	0.931	0.82	0.74	-1.122	0.309
42	MMCCYY	0.016	-1.233	0.232	0.365	1.324	-2.249
43	MMCYCY	0.014	-1.011	0.869	1.561	0.529	-2.275
44	MMCYYC	0.021	0.289	-1.3	2.714	0.362	-1.226
45	MMYCCY	0.01	-0.144	1.161	1.665	0.401	-1.961

Table S2: cont.

Sample index i	Sequence	Mean STD $\bar{\sigma}_i^E$ /-	PC1 /-	PC2 /-	PC3 /-	PC4 /-	PC5 /-
46	MMYCYC	0.021	0.166	0.998	2.17	-0.645	-0.78
47	MMYYCC	0.02	0.133	1.184	2.017	-0.853	-1.229
48	MYCCMY	0.015	0.457	0.24	-0.592	0.548	-0.611
49	MYCCYM	0.015	0.705	1.157	-0.216	-0.349	-0.552
50	MYCMCY	0.011	0.781	0.273	0.076	0.468	-1.38
51	MYCMYC	0.016	1.615	-1.218	0.355	-0.206	-0.105
52	MYCYCM	0.019	0.867	-0.182	0.942	0.45	-0.99
53	MYCYMC	0.037	1.41	-1.752	1.545	-0.388	-0.346
54	MYMCCY	0.01	0.897	1.332	0.942	-0.271	-1.118
55	MYMCYC	0.015	1.439	1.285	0.915	-1.244	0.202
56	MYMYCC	0.019	-1.816	-1.268	1.331	-0.797	0.041
57	MYYCCM	0.021	-1.24	-0.189	-0.36	-0.095	-0.797
58	MYYCMC	0.01	-1.146	-0.241	0.583	-1.42	-0.133
59	MYYMCC	0.019	-0.778	0.366	1.192	-1.431	0.072
60	YCCMMY	0.019	-0.153	0.274	-1.458	0.637	0.665
61	YCCMYM	0.023	0.077	-0.003	-1.528	0.252	0.195
62	YCCYMM	0.014	0.071	0.188	-1.48	-0.16	-0.18
63	YCMCMY	0.021	0.426	-1.641	-0.649	-0.072	1.902
64	YCMCYM	0.019	1.264	-0.956	-0.655	0.687	-0.647
65	YCMMCY	0.014	0.585	-0.054	-0.865	0.068	-0.418
66	YCMMYC	0.029	0.562	-0.164	0.689	-1.09	0.566
67	YCMYCM	0.032	0.837	-0.293	0.204	0.05	-0.688
68	YCMYMC	0.017	0.409	-0.126	1.043	-1.668	1.054
69	YCYCMM	0.027	0.91	-1.293	-1.236	0.5	-0.438
70	YCYMCM	0.014	-1.557	-1.082	-0.784	0.095	-0.598
71	YCYMMC	0.021	-1.33	-1.138	-0.317	-1.283	0.194
72	YMCCMY	0.013	-1.094	-0.009	-1.385	0.274	-0.419
73	YMCCYM	0.013	-0.752	0.983	-0.928	-0.426	-0.99
74	YMCMCY	0.014	-0.707	0.828	-0.665	-0.027	-1.454
75	YMCMYC	0.015	0.12	0.467	0.549	-0.764	-0.288
76	YMCYCM	0.02	0.168	0.17	-1.027	-0.143	-0.836
77	YMCYMC	0.019	0.386	-0.238	0.279	-0.949	-0.015
78	YMMCCY	0.013	0.337	0.911	0.241	-0.178	-1.242
79	YMMCYC	0.026	0.058	0.74	0.303	-1.25	0.047
80	YMMYCC	0.023	-0.334	0.222	1.092	-1.745	0.661
81	YMYCCM	0.013	0.167	0.56	-1.373	-0.825	0.065
82	YMYCMC	0.031	-0.179	0.479	0.009	-1.917	0.796
83	YMYMCC	0.036	-0.274	0.665	1.458	-1.613	-0.03
84	YYCCMM	0.016	-2.113	-1.135	-0.072	0.02	-0.86
85	YYCMCM	0.014	-1.944	-0.516	-0.84	-0.79	-0.596
86	YYCMMC	0.02	-0.674	-2.232	0.241	-1.124	0.783
87	YYMCCM	0.009	-0.604	-0.574	-0.435	-0.321	-0.568
88	YYMCMC	0.02	-0.305	-0.472	-0.621	-1.352	0.38
89	YYMMCC	0.021	-0.369	0.155	-0.195	-1.932	0.496

Table S3: List of labels, mean standard deviation and principal components (PCs) for every sequence in dataset 6L-PM90-$\mathcal{R}$.

Sample index i	Sequence	Mean STD $\bar{\sigma}_i^R$ /%	PC1 /-	PC2 /-	PC3 /-	PC4 /-	PC5 /-
0	CCMMYY	0.254	-1.664	-1.903	0.741	-0.103	0.211
1	CCMYMY	0.105	-1.355	-0.736	1.673	-0.427	0.194
2	CCMYYM	0.242	-1.23	0.642	1.981	-0.819	0.032
3	CCYMMY	0.188	-1.494	-0.34	1.931	-2.075	-0.504
4	CCYMYM	0.263	-1.105	1.321	1.768	-1.11	-0.368
5	CCYYMM	0.158	-0.862	2.027	1.128	-0.317	0.103
6	CMCMYY	0.287	-1.551	-1.566	0.503	0.102	0.935
7	CMCYMY	0.169	-1.549	-0.519	2.202	1.417	-0.742
8	CMCYYM	0.162	-1.135	0.953	1.223	-0.615	0.057
9	CMMCYY	0.11	-1.358	-1.563	0.203	1.672	0.068
10	CMMYCY	0.232	-0.559	-1.976	-0.806	1.298	-0.809
11	CMMYYC	0.144	0.957	-1.71	0.508	0.916	-1.048
12	CMYCMY	0.155	-0.971	-0.828	0.56	0.183	-0.784
13	CMYCYM	0.125	-0.72	0.841	0.963	0.927	-1.039
14	CMYMCY	0.173	-0.584	-0.912	-0.716	-0.165	-0.466
15	CMYMYC	0.126	0.988	-0.839	0.796	1.049	-1.028
16	CMYYCM	0.149	0.308	0.716	-0.49	-0.152	0.436
17	CMYYMC	0.186	1.208	0.338	0.727	0.616	0.296
18	CYCMMY	0.272	-1.068	-0.199	0.995	0.16	-0.946
19	CYCMYM	0.284	-0.689	1.103	0.76	-1.312	0.448
20	CYCYMM	0.216	-0.496	1.847	0.532	0.401	-0.277
21	CYMCMY	0.184	-0.667	-0.132	0.675	0.875	-0.39
22	CYMCYM	0.228	-0.523	1.414	-0.24	-0.384	0.332
23	CYMMCY	0.279	-0.542	-0.066	-1.05	0.066	-1.149
24	CYMMYC	0.231	0.957	-0.424	-0.146	-0.607	-1.68
25	CYMYCM	0.316	0.302	1.112	-0.757	0.005	0.822
26	CYMYMC	0.225	1.103	0.652	0.611	0.771	-0.446
27	CYYCMM	0.109	0.055	1.643	-0.08	1.157	1.155
28	CYYMCM	0.159	0.166	1.51	-1.047	0.582	0.068
29	CYYMMC	0.306	0.948	1.047	-0.375	0.79	-0.603
30	MCCMYY	0.442	-1.281	-1.45	0.973	1.162	1.559
31	MCCYMY	0.245	-1.201	-0.318	1.009	-0.134	0.547
32	MCCYYM	0.256	-0.957	1.149	0.985	-0.8	0.338
33	MCMCYY	0.275	-1.258	-1.368	-0.067	1.123	0.735
34	MCMYCY	0.423	-0.41	-1.595	-1.512	-0.834	1.321
35	MCMYYC	0.256	0.981	-1.46	-0.057	0.39	-0.315
36	MCYCMY	0.236	-0.69	-0.436	0.307	-0.328	1.478
37	MCYCYM	0.481	-0.7	0.88	-0.388	-0.927	0.949
38	MCYMCY	0.245	-0.469	-0.469	-1.382	-1.343	1.57
39	MCYMYC	0.146	1.124	-0.682	0.462	-1.053	1.087
40	MCYYCM	0.382	0.284	0.695	-0.472	-0.673	2.576
41	MCYYMC	0.238	1.012	0.388	-0.485	-0.292	1.505
42	MMCCYY	0.521	-1.095	-1.015	-0.949	0.363	2.513
43	MMCYCY	0.315	-0.291	-1.184	-1.132	1.879	1.573
44	MMCYYC	0.186	0.925	-1.268	-1.096	-0.136	0.191
45	MMYCCY	0.273	-0.057	-1.301	-1.736	-0.342	1.786

Table S3: cont.

Sample index i	Sequence	Mean STD $\bar{\sigma}_i^R$ /-	PC1 /-	PC2 /-	PC3 /-	PC4 /-	PC5 /-
46	MMYCYC	0.266	1.34	-1.237	-0.047	0.533	0.123
47	MMYYCC	0.166	1.837	-1.005	1.417	0.556	0.972
48	MYCCMY	0.118	-0.356	-0.455	-0.193	1.024	1.558
49	MYCCYM	0.155	-0.378	0.991	-0.07	0.111	0.951
50	MYCMCY	0.123	-0.183	-0.524	-0.855	1.293	0.734
51	MYCMYC	0.216	1.232	-0.774	0.147	-0.099	0.267
52	MYCYCM	0.178	0.259	0.314	0.036	-1.647	1.24
53	MYCYMC	0.171	1.109	-0.284	0.777	-1.717	-0.05
54	MYMCCY	0.094	-0.582	-1.051	-1.205	-0.643	-1.134
55	MYMCYC	0.204	1.089	-1.257	0.546	-1.529	-1.014
56	MYMYCC	0.134	1.852	-0.882	2.333	-0.718	0.212
57	MYYCCM	0.193	0.618	0.392	0.617	0.54	0.913
58	MYYCMC	0.104	1.341	-0.35	0.814	-1.265	0.794
59	MYYMCC	0.162	1.756	-0.097	1.892	0.309	1.12
60	YCCMMY	0.295	-1.225	-0.602	0.73	0.109	-1.084
61	YCCMYM	0.185	-0.979	1.022	0.742	-0.19	-1.398
62	YCCYMM	0.277	-0.912	0.961	0.291	0.201	-1.387
63	YCMCMY	0.549	-0.973	0.126	-0.932	-0.381	-0.929
64	YCMCYM	0.159	-0.919	0.981	-0.561	-1.443	-0.836
65	YCMMCY	0.153	-0.818	-0.543	-1.567	-0.291	-2.015
66	YCMMYC	0.184	0.945	-0.736	-0.274	-1.452	-2.053
67	YCMYCM	0.171	0.252	0.939	-0.494	0.265	-0.112
68	YCMYMC	0.176	1.059	0.401	0.047	-0.352	-0.62
69	YCYCMM	0.158	-0.169	1.401	-0.516	-0.248	1.055
70	YCYMCM	0.293	-0.083	0.955	-1.961	-1.623	0.37
71	YCYMMC	0.141	1.086	0.817	-0.203	0.351	0.5
72	YMCCMY	0.24	-1.196	-0.393	-0.931	0.474	-1.373
73	YMCCYM	0.272	-0.955	1.063	-1.295	-1.556	-0.22
74	YMCMCY	0.372	-0.673	-0.438	-1.654	-0.068	-0.853
75	YMCMYC	0.392	0.912	-0.829	-0.722	-1.289	-1.853
76	YMCYCM	0.265	0.207	1.069	-1.246	-0.228	0.114
77	YMCYMC	0.232	0.937	0.34	-1.115	-1.876	0.014
78	YMMCCY	0.476	-0.69	-0.361	-2.119	0.398	-1.769
79	YMMCYC	0.353	1.048	-0.501	-0.929	-1.392	-0.635
80	YMMYCC	0.213	1.693	-0.505	0.677	-0.818	-0.761
81	YMYCCM	0.162	0.47	0.857	-0.771	0.669	0.57
82	YMYCMC	0.134	1.336	0.498	0.214	1.001	-0.72
83	YMYMCC	0.169	1.697	0.571	1.102	1.334	-0.055
84	YYCCMM	0.29	-0.097	1.626	-0.146	2.834	-0.305
85	YYCMCM	0.185	0.115	1.313	-0.873	1.869	-0.476
86	YYCMMC	0.467	1.04	0.803	-0.54	1.897	-0.926
87	YYMCCM	0.357	0.297	1.289	-0.887	1.763	-0.377
88	YYMCMC	0.093	1.367	0.01	0.175	0.12	-0.45
89	YYMMCC	0.088	1.504	0.063	0.315	0.221	-0.398
